# Pharmacology of Catechins in Ischemia-Reperfusion Injury of the Heart

**DOI:** 10.3390/antiox10091390

**Published:** 2021-08-30

**Authors:** Kristína Ferenczyová, Lucia Kindernay, Jana Vlkovičová, Barbora Kaločayová, Tomáš Rajtík, Monika Barteková

**Affiliations:** 1 Centre of Experimental Medicine, Institute for Heart Research, Slovak Academy of Sciences, Dúbravská cesta 9, 84104 Bratislava, Slovakia; kristina.ferenczyova@savba.sk (K.F.); lucia.griecsova@savba.sk (L.K.); jana.vlkovicova@savba.sk (J.V.); barbora.kalocayova@savba.sk (B.K.); 2 Department of Pharmacology and Toxicology, Faculty of Pharmacy, Comenius University in Bratislava, Odbojárov 10, 83232 Bratislava, Slovakia; rajtik@fpharm.uniba.sk; 3 Institute of Physiology, Comenius University in Bratislava, Sasinkova 2, 81372 Bratislava, Slovakia

**Keywords:** heart, catechins, antioxidants, ischemia-reperfusion (I/R) injury, myocardial infarction, cardioprotection, RISK pathway, JNK pathway, apoptosis, microRNAs (miRs)

## Abstract

Catechins represent a group of polyphenols that possesses various beneficial effects in the cardiovascular system, including protective effects in cardiac ischemia-reperfusion (I/R) injury, a major pathophysiology associated with ischemic heart disease, myocardial infarction, as well as with cardioplegic arrest during heart surgery. In particular, catechin, (−)-epicatechin, and epigallocatechin gallate (EGCG) have been reported to prevent cardiac myocytes from I/R-induced cell damage and I/R-associated molecular changes, finally, resulting in improved cell viability, reduced infarct size, and improved recovery of cardiac function after ischemic insult, which has been widely documented in experimental animal studies and cardiac-derived cell lines. Cardioprotective effects of catechins in I/R injury were mediated via multiple molecular mechanisms, including inhibition of apoptosis; activation of cardioprotective pathways, such as PI3K/Akt (RISK) pathway; and inhibition of stress-associated pathways, including JNK/p38-MAPK; preserving mitochondrial function; and/or modulating autophagy. Moreover, regulatory roles of several microRNAs, including miR-145, miR-384-5p, miR-30a, miR-92a, as well as lncRNA MIAT, were documented in effects of catechins in cardiac I/R. On the other hand, the majority of results come from cell-based experiments and healthy small animals, while studies in large animals and studies including comorbidities or co-medications are rare. Human studies are lacking completely. The dosages of compounds also vary in a broad scale, thus, pharmacological aspects of catechins usage in cardiac I/R are inconclusive so far. Therefore, the aim of this focused review is to summarize the most recent knowledge on the effects of catechins in cardiac I/R injury and bring deep insight into the molecular mechanisms involved and dosage-dependency of these effects, as well as to outline potential gaps for translation of catechin-based treatments into clinical practice.

## 1. Introduction

Ischemia-reperfusion (I/R) injury of the heart represents a major cardiovascular pathology associated with such clinically relevant states as coronary ischemic heart disease and acute myocardial infarction, as well as with cardioplegic arrest during heart surgery or transplantation. One of the key mechanisms involved in the development of I/R injury is oxidative stress (OxS) due to redox dysbalance within cardiac tissue associated with excessive production of reactive oxygen and nitrogen species (ROS, RNS), mitochondrial dysfunction, and apoptosis [1,2]. Thus, potential cardioprotective strategies targeting OxS and redox homeostasis-associated molecular pathways have been extensively studied in cardiac I/R injury aimed to prevent the heart from I/R-induced cell damage and improve cardiac function post I/R. In line with this aim, numerous compounds with antioxidant properties have been evaluated in cardiac I/R injury, including natural and synthetic antioxidants, their combinations, and antioxidant-rich extracts and mixtures [3,4,5].

Polyphenols represent a big superfamily of natural substances with antioxidant properties that can be divided into several sub-families, including flavonoids, stilbenes, lignans, and phenolic acids, all possessing strong antioxidant properties and suggested to be beneficial in many human diseases [6].

Catechins belong to the flavonoid subgroup of polyphenols that involves structurally related compounds, including (+)-catechin, (−)-epicatechin (EPI), (−)-epigallocatechin (EGC), (−)-epicatechin gallate (ECG), and (−)-epigallocatechin gallate (EGCG). Catechins possess strong antioxidant properties and are suggested to have certain therapeutic potential in several diseases associated with OxS, including neural and cardiometabolic diseases [7,8] or cancer [9]. The main dietary sources of catechins are tea and cocoa, but also beans and fruits, such as cherries, grapes, apricots, peaches, blackberries, and apples [10].

In the cardiovascular system, catechins have been shown to exert effective antihypertensive effects in subjects with different stages of hypertension documented in animal studies [11,12]. On the other hand, several clinical studies in human patients with different degrees of blood pressure elevation brought both positive and neutral results regarding potential antihypertensive effects of catechins and catechin-rich food or extracts [13,14]. Catechins were also reported to exert vasculoprotective effects and to prevent endothelial dysfunction in both pre-clinical experimental studies [11] and clinical trials [14,15]. In addition to the above-mentioned beneficial effects of catechins on the vasculature, protective effects of various catechin compounds and catechin-rich foods and extracts were reported in I/R injury of the heart, mainly documented in animal studies as well as in in vitro cell-based models of cardiac I/R injury, thus, suggesting promising cardioprotective potential of catechins for the management of patient suffering from ischemic heart disease or myocardial infarction. Although beneficial effects of catechins in different human diseases, including cardiovascular, have been recently reviewed [7,16], the effects of catechins, particularly in myocardial I/R injury, have not been reviewed so far. Thus, the aim of this focused review is to summarize the recent knowledge on the beneficial effects of catechins in cardiac I/R injury and to bring deep insights into the pharmacology of catechins in this cardiac pathology, including molecular mechanisms involved in their action, as well as dose- and treatment-duration-dependency of catechin effects in cardiac I/R injury. Potential clinical implications of catechins in human I/R heart injury and translational gaps will be discussed as well.

## 2. Chemical Structure, Features, Sources, and Stability of Catechins

Catechins are natural polyphenols with typical core structure sharing a common backbone with other bioactive flavonoids. Catechins belong to flavanols, a subclass derived from 2-phenylbenzopyran-based flavonoids that are widely represented in plant kingdom as secondary metabolites. The most studied are tea catechins, including (−)-catechin, EPI, ECG, and EGCG, but also other catechins, such as procyanidins, e.g., (+)-cyanidanol [17,18,19].

**Figure 1:**
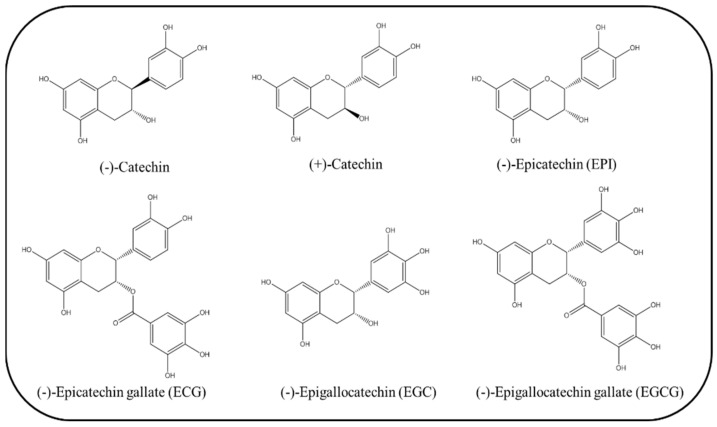
The chemical structures of major catechin derivatives.

A common chemical feature shared in tea catechins is a benzene ring (A) joined to dihydropyran heterocycle with hydroxyl group in C3 position (C) and a pyrogallol ring (B). Thanks to C3 hydroxyl group, catechins are also known as flavan-3-ols. On the same hydroxyl group, an important chemical reaction occurs—esterification leading to the existence of various catechin gallates [20]. Catechins exist not only in the form of monomers but also in the form of oligomers called pro-anthocyanidins. In their pure state, catechins are crystalline and colorless compounds with hydrophilic properties. Typical representative of the green tea catechin family with no side group in its molecule is (−)-catechin (Figure 1). Other catechins derived from tea are characterized by different side groups in various positions (substituents or hydroxyl groups placed on the rings or on the galloyl group ((D or B’), Figure 1). All these chemical features determine biological activity of individual catechins, as well as their ability to interact with the surrounding environment [21].

The health benefits attributed to green tea manufactured from the leaves of the *Camellia sinesis* plant relate to high proportion of polyphenol flavonoids, which are maintained in high concentrations [22]. Catechins in the final tea products can be preserved due to deactivation of endogenous polyphenol oxidase (PPO) during the steaming process or can be degraded by a so-called “fermentation” process, due to which catechins undergo extensive oxidation by two enzymes—PPO and/or peroxidase. Therefore, depending on the environment, plucking period, and manufacturing process (whether it is green tea, black tea, white tea, or oolong tea), catechins can stay maintained in the final product in various amounts [23,24]. Catechins are the main phenolic substances enriched in green tea in the range from 80 to 90%, but only 20 to 30% in black tea due to fermentation process, where, on the other hand, theaflavins, thearubigins, and theasinensins are in more excess [25,26,27]. Thus, the main determinant of the final amount of catechins in each type of tea is the manufacturing process. Whereas black tea requires full fermentation of the fresh plant leaves and oolong tea partial fermentation, production of green tea involves only steaming and drying [28]. White tea is a special category itself. In general, it is considered a “green tea” sort of tea, but it is grown in specific places, e.g., Fujian Province. Moreover, the term “white tea” is derived from its production completely from leaf buds covered with white and downy hairs [29].

Major representatives of the catechin family in various teas are EGCG, ECG, EGC and EPI (Figure 1). The most studied and biologically active catechin with great therapeutic potential is EGCG, which is also in abundance (59%) in green tea compared to other catechins [30]. It was shown that the biological potential of EGCG is derived from a large number of active phenolic hydroxyl groups and structure moiety [31]. Other natural, edible, and quite common sources of flavan-3-ols and pro-anthocyanidins are cocoa beans (mainly EPI), berries, nuts, and beans [32].

## 3. Metabolization of Catechins in the Human Body

Extensive studies have demonstrated that catechins are unstable under physiologic conditions and have relatively low bioavailability determined by their poor systemic absorption in the gastrointestinal tract after oral treatment but also due to their rapid elimination from the blood when administered intravenously. This leads to the low level of catechins in systemic circulation or tissues [33]. However, different bioavailability studies of catechins used different approaches, methodologies, and conditions (dosage, treatment, time of administration); therefore, to date, there is no generally accepted consensus on the level of bioavailability of catechins. Gut microbiota appears to be essential for catechins absorption; therefore, it can probably play a pivotal role in the proven health beneficial effects of orally administered catechins [18,24,34].

The Lipinski rule explains the hypothetically low bioavailability of catechins due to their high molecular weight (300–450 kDa) and due to the presence of five and more hydroxyl groups attached to the backbone [35]. In vitro studies showed low absorption of catechins after oral pre-treatment moving in range 5–50 times less in plasma than is needed to exert biological activities [33,36]. Based on in vitro studies, it seems that human and mice might be closer to each other in absorption of EGCG than either of them to rats in terms of extensive glucuronidation of EGCG in mouse and human liver compared to higher proportion of methylation and sulfation in rat liver [37,38]. The major barrier for catechin absorption seems to be the small intestine due to the higher pH inside it [39,40]. After oral administration, the peak blood concentration of EGCG varies within 1 to 2 h after ingestion, i.e., when absorbed by the small intestine [41], and the peak plasma concentrations of EGCG after oral treatment were very low in comparison to the applied dose of EGCG or catechin rich extract, e.g., green tea [36,42,43,44,45,46].

**Figure 2:**
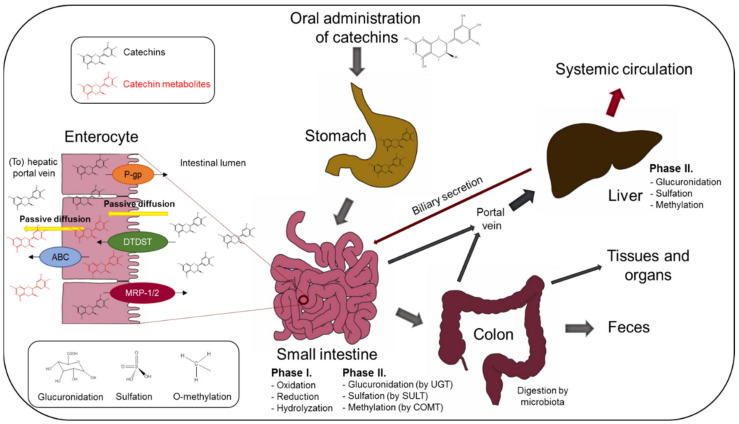
Metabolization of catechins in the body. After oral ingestion, catechins pass downstream via the organs of the gastrointestinal tract, including stomach, small intestine, liver (by portal vein), and colon. In the process of systemic biotransformation, catechins are metabolized by enzymes of phase I and phase II metabolism in small intestine and/or liver and by gut-microbiota in colon, causing modifications to have higher bioavailability. In the small intestine, catechins are transported from intestinal lumen to enterocytes mostly by passive diffusion and undergo phase I and II enzyme modifications. Recently, a novel transporter of EGCG, DTDST, was identified [47]. Two known transporters are responsible for lower bioavailability of catechins by transferring them from enterocyte back to intestinal lumen, P-gp and MRP-1/2. Otherwise, catechins and catechin metabolites continue from enterocytes by passive or active transport (by ABC transporters) via hepatic portal vein to liver and further to systemic circulation, or they are secreted back to the small intestine by bile. A fraction of unprocessed catechins, catechin metabolites, and catechin oligomers or polymers undergo final metabolization in the colon by gut-microbiota or are unabsorbed and excreted by feces. Degradation by microbiota leads to formation of small absorbable phenolic acids transported to tissues and organs, where they perform their biological activities. Abbreviations: P-gp, P-glycoprotein (ATP-dependent protein pump); MRP-1/2, multidrug resistance-associated protein 1 or 2; DTDST, diastrophic dysplasia sulfate transporter; ABC, ABC transporter; UGT, UDP-glucuronosyltransferase; SULT, sulfotransferase; COMT, catechol-O-methyltransferase.

After oral administration, catechins pass through the stomach and later, together with gastric juice, move to the duodenum, where it is neutralized by bicarbonate ions. While the stomach provides a stable environment for EGCG, EGCG is not stable in the small intestine due to higher pH [31]. In the lumen of the small intestine, the absorption of catechins begins by entering enterocytes where certain portion of catechins may undergo a phase I metabolism (oxidation, reduction, and hydrolyzation). For a long time, no specific transporters have been identified to carry catechins through the enterocyte, but recently, Ishii et al. [47] discovered diastrophic dysplasia sulfate transporter (DTDST) as a novel potential EGCG transporter in the ileum that is upregulated after repeated oral catechin administration. However, it is still suggested that catechins are transported preferably by passive diffusion by maintaining the concentration gradient using both transcellular and paracellular ways. EGCG and its metabolites are absorbed back into intestinal space by two efflux ATP-dependent protein pumps—P-glycoprotein (P-gp) or by multidrug resistance-associated protein 1 or 2 (MRP-1 or 2)—resulting in its lower bioavailability [48,49,50,51]. Studies in Caco-2 monolayer cells demonstrated that not only ungallated catechins [52,53] but also EGC, ECG, and EGCG (with lower affinity) are transported by MRPs [24,54]. In enterocytes, a part of catechins undergo a phase II metabolism reaction catalyzed by conjugation enzymes (sulfate conjugation and/or glucuronidation), specifically by SULT (sulfotransferase), UGT (UDP-glucuronosyltransferase), and by modification enzyme COMT (catechol-O-methyltransferase). While sulfation and glucuronidation enhance hydrophilic properties of catechins to increase their solubility and preferably occur in the small intestine, methylation, together with sulfation and glucuronidation, occurs in the liver. A small fraction of catechins remains in non-conjugated free form and enters the small intestine and downstream organs. Catechins are further transported either in the free form or in the form of water-soluble metabolites (sulfate, glucuronide, and methyl derivatives) by portal vein to the liver, where they are further metabolized by phase II metabolism enzymes (SULT, UGT, COMT) and excreted by the bile back to the duodenum or to systemic circulation. Unabsorbed catechins, catechin metabolites, and catechin oligomers or polymers pass straight into the colon from secreted bile from portal circulation, where they undergo the final deconjugation by microorganisms or are excreted by urine or feces [33,55,56,57]. Thus, degradation leads to formation of small products of catechins catabolism—absorbable phenolic acids distributed to various tissues and organs, where they can be further metabolized or perform their biological activity. In fact, most of the tea catechins perform their biological activities via phenolic acids because of their limited bioavailability in the small intestine after oral administration [34]. The broad spectrum of microbiota that inhabits the colon is disposed with many different enzymes catalyzing decomposition of incoming nutrition in the large intestine. Microbial culture is essential for the host in terms of metabolism, digestion, catabolism, and overall health. Hence, a set of enzymes derived from gut microbiota can perform many different types of chemical reactions, in particular, demethylation, dihydroxylation, isomerization, decarboxylation, reduction, hydrolyzation, non-aromatic ring cleavage, etc. [24,34,58,59,60,61]. It is also suggested that the resulting metabolites of catechin depend on the method of application. [61,62,63,64]. All metabolites originating in the large intestine are transported by portal vein to the liver, where Phase II metabolism is taking place. After all, these metabolites can enter systemic circulation and perform their biological activities. Subsequently, they are absorbed and excreted by urine, or they are unabsorbed and excreted in feces [34,44,65,66]. The overview of the metabolization of catechins in the body is presented in the Figure 2.

## 4. Effects of Catechins on Cardiac Function, Cell Viability, and Infarct Size in Myocardial I/R Injury

Post-ischemic recovery of cardiac function and the size of infarction represent the most relevant outcomes of studies revealing the effects of any cardioprotective interventions including catechins in in vivo and ex vivo animal models of myocardial infarction/cardiac I/R injury. In in vitro studies, the most relevant outcome is measuring the cell viability after simulated ischemia- or hypoxia-induced injury in cardiac-derived cell lines or primary cardiomyocyte cultures. In some studies, the cardioprotective effects are documented by indirect markers, e.g., by monitoring release of cardiac-injury-related enzymes, such as LDH, TnI, CK-MB, as well as by monitoring apoptosis rate. Particularly, in some in vitro studies, the apoptosis rate is presented as the main outcome assessing the extent of the injury. However, cardiac function, cell viability, and infarct size (IS) are considered the most relevant parameters to evaluate cardiac damage, and these parameters are the best predictors of cardiac recovery after ischemic insult. Thus, here, we summarize the effects of catechins on these relevant parameters documented in different in vitro, ex vivo, and in vivo models of cardiac I/R injury.

### 4.1. Pre-Clinical In Vitro Cell-Based Studies

Several in vitro studies documented effects of catechins on hypoxia- or anoxia-induced loss of cell viability in cardiac-derived cell cultures. Cell viability in these studies was measured using either CCK-8 (Cell Counting Kit-8) assay or MTT (3-[4,5-dimethylthiazole-2-yl]-2,5-diphenyltetrazolium bromide) assay. In H9c2 cardiomyoblast cell line, catechin administered in different concentrations (5–50 μM) into the culture medium before H/R induction significantly increased cell viability (detected by CCK-8) after H/R. The improved cell viability was associated with improved mitochondrial function and reduced apoptosis, likely through regulating CREB/lncRNA MIAT/Akt/Gsk-3β pathway [67]. Furthermore, catechin in the 100 or 200 μM concentration protected H9c2 cells from hypoxia-induced cell viability loss, inhibition of cell proliferation, and apoptosis, likely by regulating miR-92a and JNK signaling pathway [68]. A study performed in cultured neonatal mouse cardiomyocytes (NMCMs) demonstrated that EPI in the 5 µM concentration improved cell viability (measured by MTT) of NMCMs exposed to 12-h anoxia and reduced apoptosis via activation of the PTEN/PI3K/Akt pathway [69]. Improved cell viability due to treatment with catechins has been documented also in cultured primary neonatal rat cardiomyocytes (NRCMs). Particularly, EGCG increased the survival rate and spontaneous beating of NRCMs during reoxygenation after 3h hypoxia, decreased the LDH and MDA release, and increased SOD and ATP enzyme activities, likely via scavenging of free radicals and PKC/Gi signal transduction [70]. EGCG in 10 μM concentration was shown to improve cell viability (measured by MTT) and reduce apoptosis in H9c2 cells exposed to H/R injury along with improved mitochondrial function [71]. EGCG in different concentrations (6.25–25 μM) improved cell viability of H9c2 cells after H/R-injury along with attenuating mitochondrial impairment and apoptosis by regulation of miR-30a/p53 axis [72]. Another study, however, did not show improvement of cell viability (by CCK-8) due to EGCG treatment (25 µM) in H9c2 cells exposed to H/R, but EGCG exerted a protective role in microRNA-384-mediated autophagy by targeting Beclin-1 via activating the PI3K/Akt signaling pathway in this study [73]. EGCG preserved cell viability (measured by MTT) also in H/R-challenged H9c2 cells under hyperglycemic conditions through decreased apoptosis and OxS by stimulating the SIRT1 signaling pathway [74]. Very recently, exosomes derived from EGCG-treated H9c2 were shown to improve H9c2 cell viability (measured by CCK-8) of H/R-challenged H9c2 cells via increasing microRNA-30a, along with inhibiting apoptosis and excessive autophagy [75]. As mentioned, some in vitro studies did not reveal cell viability after H/R but reported cardioprotective effects of catechin-based treatments documented by reduced apoptosis rate or improved mitochondrial function as the main outcomes in these studies [76,77]. However, it might be questionable whether these parameters are direct indicators of cardiomyocyte survival and function or should be considered just the mechanisms underlying cardioprotective effects of catechins and catechin-based combined therapies.

### 4.2. Pre-Clinical Ex Vivo Studies in Isolated Heart

In pre-clinical studies documenting effects of catechins in ex vivo models of myocardial I/R injury, two types of experimental design were used: (i) ex vivo application of catechins directly into the perfusion medium for perfusion of the heart exposed to ex vivo I/R and (ii) in vivo application of catechins to experimental animals combined with ex vivo I/R in isolated hearts after sacrificing the animals.

Early studies documented beneficial effects of catechins on post-ischemic recovery of the heart function, mostly in pure ex vivo studies. For example, van der Kraaij et al. documented that (+)-catechin (also referred as (+)-cyanidanol) dissolved in the 20 μM concentration in the perfusion solution and present throughout the whole experiment significantly improved post-ischemic recovery of contractile function, coronary flow, and prevented ventricular arrhythmias in isolated rat hearts exposed to either anoxia/reperfusion (A/R, 45 min/20 min) or I/R (15 min/15 min) in normal, iron-loaded, as well as iron-chelator, co-treated rats. This protection of heart function was associated with decreased LDH release but unchanged activities of SOD and glutathione peroxidase (GPx) [78,79]. Similar beneficial ex vivo results, i.e., improved post-ischemic cardiac performance, were obtained in isolated rabbit hearts exposed to 40 min low-flow ischemia/20 min reperfusion with 100/200 μM concentration of procyanidins isolated from *vitis vinifera* seeds in the perfusion solution, likely via scavenging of free radicals and interaction with metal ions [80,81], as well as in isolated rat hearts exposed to global I/R (30 min/30 min) with 100 μM catechin in the perfusion solution [82]. Furthermore, ex vivo application of EGCG (3 × 10^−5^ M) or (−)-gallocatechin-3-gallate (GCG, 3 × 10^−6^ M) improved post-ischemic recovery of cardiac performance in isolated guinea pig hearts exposed to global I/R (40 min/40 min), and this protection was associated with preserved levels of ATP, inhibited mitochondrial Ca^2+^ elevation, and decreased ROS and caspase-3 activity [83]. Improved heart function was documented also in the rabbit model of cardioplegic arrest (global I/R for 90 min/60 min) with 20 µM EGCG in the cardioplegic solution, likely via preserved ATP levels, decreased HSP60, and decreased nitrosative/oxidative stress [84]. In addition to improving cardiac contractile function, studies documented also positive effects of ex vivo catechin administration on IS. For example, 1 or 10 µM of EGCG applied at different times before/during/after ischemia significantly reduced IS in isolated rat hearts subjected to regional I/R (30 min/2 h) [85,86,87], and the same compound (EGCG) in the 5 µM concentration in the perfusion solution reduced IS in isolated rat hearts exposed to global I/R (20 min/2 h), along with improved cardiac function and decreased levels of LDH, likely via increased endogenous antioxidant enzymes (MnSOD, catalase) and reduced apoptosis rate [88]. In higher 100 µM concentrations, EGCG infused into the perfusion solution 30 min before 35 min regional ischemia/120 min reperfusion reduced IS and improved post-ischemic ventricular function of isolated rat hearts, likely via reduction of apoptosis after inhibition of p-STAT-1 [89]. A similar reduction of IS was documented also in isolated rat hearts treated with different concentrations (10, 50, 100, or 500 ng/mL) of ECG applied into the perfusion solution for 4 min before I/R (30 min/2 h) with 8 min washout or during initial 4 min of reperfusion. This IS reduction was accompanied with improved recovery of cardiac function after I/R, improved function of Na/K-ATPase, opening of mitochondrial KATP channels, and activation of PKC-ε [90]. Very recently, the cardioprotective effect of EGCG directly applied to the perfusion solution (10 mg for 30 min prior to I/R) was confirmed in isolated rat hearts exposed to ex vivo global I/R (30 min/120 min), and this was manifested by significantly reduced IS accompanied by increased Ca^2+^ and decreased TnT in the coronary perfusion fluid [91].

In combined studies using in vivo catechin application and consequent ex vivo myocardial I/R in isolated hearts, it was documented that catechin (250 mg/kg) administered intragastrically, either as a single dose 60 min before the sacrifice of rats or for 10 days before the sacrifice of rats, significantly improved recovery of cardiac function of isolated hearts subjected to ex vivo I/R (30 min/30 min), and this cardioprotection was associated with decreased LDH release and lipid peroxidation [82]. Further, procyanidins isolated from *vitis vinifera* seeds mixed with food at 2.4% *w/w* and applied to rats for 3 weeks improved recovery of cardiac function of isolated hearts exposed to ex vivo I/R by 20 min of low-flow ischemia/30 min reperfusion. This protective effect was associated with enhanced total antioxidant plasma capacity and the plasma levels of ascorbic acid [92]. The catechin derivative EGCG in the 0.1, 1, or 10 mM concentration applied in vivo to rats for 2 weeks orally (in drinking solution) improved functional recovery of isolated rat hearts exposed to 30 min global ischemia/60 min reperfusion, likely via decreasing OxS and apoptosis rate, although a high dose did not lead to dramatic improvement in cardiac function [93]. Finally, EGCG in the dose 200 mg/kg/day applied for 3 weeks in drinking water to spontaneously hypertensive rats (SHR) significantly improved cardiac function and reduced IS in isolated hearts subjected to ex vivo I/R (30 min/2 h), suggesting cardioprotective potential of catechins also in the presence of comorbidities (hypertension) [94].

### 4.3. Pre-Clinical In Vivo Animal Studies

The vast majority of preclinical studies examined effects of catechins on cardiac function and IS in in vivo models of cardiac I/R injury, primarily in left anterior descending (LAD) coronary artery occlusion-induced MI and, to a lower extent, in isoproterenol (ISO)-induced MI. Most of these studies revealed effects of two major catechin derivatives, EPI and EGCG, in rodents. A few studies revealed in vivo effects of other catechins, e.g., catechin or procyanidin, and only rarely were the effects of catechins revealed in large animals. It was documented that EPI in a dose of 1 mg/kg/day administered via oral gavage to male rats for 10 days significantly reduced IS, but shorter, 2-day pretreatment, in the same dose did not reduce IS. Heart hemodynamics were not affected with both 2- and 10-day treatment, but EPI treatment in both durations significantly reduced heart tissue OxS in the study [95]. The same oral dose of EPI (1 mg/kg/day for 10 days) reduced IS in mice subjected to in vivo I/R (30 min/2h), likely via δ-opioid receptor activation [96], as well as in rats exposed to permanent coronary artery occlusion [97]. The same dose of EPI improved heart function during in vivo persistent ischemia for 7 days in mice, along with reduced cardiac fibrosis, hypertrophy, and apoptosis, likely via activation of the PTEN/PI3K/AKT pathway [69]. A higher, 10 mg/kg dose of EPI applied alone or in combination with doxycycline in a single dose intravenously, reduced IS in rats exposed to 45 min LAD occlusion and different times of reperfusion [98,99]. In addition, EPI applied intragastrically in the dose 20 mg/kg/day for 21 days improved cardiac function and reduced IS in ISO-induced MI in rats, along with reduced necrosis and inflammation, likely via decreasing OxS [100,101]. A 10-day oral pretreatment with 250 mg/kg/day of catechin improved heart function and reduced IS in rats subjected to I/R (30 min/24 h) via downregulation of lncRNA MIAT expression in heart tissue [67].

The most extensively studied catechin derivative, EGCG, applied intragastrically to rats (50 mg/kg/day) for 7 weeks after induction of LAD occlusion improved cardiac function and prevented cardiac remodeling via inhibition of dual-specificity tyrosine phosphorylation regulated kinase 1A/alternative splicing factor/calcium/calmodulin dependent protein kinase IIδ (Dyrk1A/ASF/CaMKIIδ) pathway [102]. Intravenous administration of EGCG (10 mg/kg) before reperfusion reduced IS in rats exposed to I/R (30 min/2 h) through activation of RISK survival pathways [103]. EGCG (10 or 20 mg/kg) injected into sublingual veins of rats at different times before LAD occlusion or before the onset of reperfusion reduced IS and improved cardiac function; reduced necrosis, apoptosis, and inflammation; and improved cardiac ultrastructure via reduced autophagy and modulation of several miRNAs [72,73,75,104]. EGCG injected into femoral vein before in vivo I/R (30 min/2 h) significantly reduced IS and improved the ultrastructure of cardiomyocytes [91]. Notably, improved cardiac function and reduced IS was documented after 2-week oral treatment with EGCG (100 mg/kg/day) in STZ-induced diabetic rats exposed to in vivo cardiac I/R (30 min/2 h), suggesting cardioprotective potential of catechins also in the presence of metabolic comorbidities [74]. On the other hand, in a rare study performed in large animals, EGCG (10 mg/kg) applied intravenously to domestic pigs before in vivo I/R (90 min/2 h) did not improve cardiac function post I/R, despite a reduction of markers of myocardial damage, suggesting a translational gap of catechin treatments from rodents to large animals and, potentially, to humans [105]. The overview of catechin effects on IS, cardiac function, and cell viability is presented in Table 1.

### 4.4. Clinical/Human Studies

Cardiovascular effects of catechins and catechin-enriched foods were documented in several clinical studies. In studies focused on the effects of catechins on blood pressure, controversial findings were observed. While some studies reported beneficial effects of these compounds on blood pressure (antihypertensive effects), e.g., the recent clinical trial by Chatree et al. [106], other studies revealed no beneficial effects of catechins or catechin-rich food on blood pressure [107,108]. Some studies also revealed the effects of catechin intake on the cardiovascular and cardiometabolic risk of different patient cohorts, and these studies also brought controversial results, both positive [109,110] and neutral (no reduction of CVD risk) [108]. However, regarding the particular focus of this article, clinical studies documenting direct effects of catechins or catechin-rich foods on cardiac I/R injury in humans, i.e., in ischemic heart disease, MI, heart surgery, or transplantation, have not been published so far. Thus, taking together the inconclusive results of existing clinical trials of catechins in any CVDs and the lack of human studies directly focused on the effects of catechins in ischemic heart disease, it should be concluded that a significant translational gap exists in transferring preclinical experimental knowledge of effects of catechins on cardiac function and cardiomyocyte survival in I/R to the real benefits for human patients suffering from ischemic heart disease or MI or those undergoing heart surgery.

## 5. Role of Pro/Antioxidant Balance and Redox Signaling in Catechin Effects in Cardiac I/R

Since oxidative stress (OxS) represents one of the major mechanisms underlying cardiac I/R injury (for review see: [2,111]), compounds with antioxidant properties are widely examined for their cardioprotective effects in this pathology. In line with this, catechins were primarily used in cardiac I/R injury as effective antioxidants targeting OxS and redox signaling in this cardiac pathology. Therefore, in this chapter we summarize effects of catechins on redox homeostasis, cardiac antioxidant defense enzymes, and intracellular redox signaling pathways associated with their beneficial effects in myocardial I/R injury.

### 5.1. Direct and Indirect Antioxidant Effects of Catechins in Myocardial I/R Injury

Redox homeostasis in the heart tissue under both physiological and pathological conditions is maintained by the balance between pro-oxidants, mainly free radicals produced by mitochondrial cytochromes, xanthine oxidoreductase, nicotinamide adenine dinucleotide phosphate (NADPH) oxidase and nitric oxide synthase (NOS), and antioxidant defense enzymes, including superoxide dismutase (SOD), catalase (CAT), and glutathione peroxidase/reductase (GPx/GRx). Excessive free radical production in I/R leads to the oxidative damage to several types of cardiac molecules, including lipid peroxidation, and protein and DNA alterations, finally, leading to cardiac dysfunction and cardiomyocyte cell death [2]. Catechins, in addition to their direct antioxidant effects, have been documented to execute their beneficial effects in cardiac I/R injury also via activating endogenous antioxidant enzymes, thus, acting as indirect antioxidants as well.

As for the direct antioxidant effects of catechins in cardiac I/R, levels of OxS in the heart tissue were evaluated either by measuring intracellular reactive oxygen species (ROS) levels or by measuring levels of OxS markers, including thiobarbituric acid reactive substances (TBARS) or 8-hydroxy-2’-deoxyguanosine (8-OHdG). Regarding direct radical-scavenging action of catechins in myocardial I/R injury, it was documented that procyanidines from *vitis vinifera* seeds are potent scavengers of several ROS involved in the I/R-induced cardiac damage, including the superoxide anion (determined by the phenazine methosulfate/NADH method), the hydroxyl radical (determined by the electron spin resonance spectroscopy), and peroxyl radicals (determined using UV spectroscopy). Moreover, they interacted with Fe^2+^ and Cu^2+^ ions, the catalysts of production of HO·radicals. These antioxidant effects of procyanidines were associated with improved cardiac function in isolated rabbit hearts exposed to 40min low flow ischemia [80]. Reduced cardiac OxS (measured by TBARS levels) due to in vivo EPI or EGCG treatment was documented in ISO-induced MI in rats [100,112,113,114]. Reduced OxS (documented by reduced 8-OHdG) was reported also in a combined model of 2-week in vivo EGCG treatment with subsequent ex vivo I/R in isolated rat hearts [93]. Very recently, decreased intracellular levels of ROS due to treatment with catechin was associated with improved mitochondrial membrane potential, decreased apoptosis, and increased cell survival after H/R in H9c2 cells [67]. Finally, EGCG significantly reduced formation of PAR (poly-ADP-ribose) and NT (nitrotyrosine), the markers of oxidative and nitrosative stress, in the heart tissue during cardioplegia in isolated perfused rabbit hearts [84].

Several studies documented the protective effects of catechins on myocardial lipid peroxidation in I/R injury, commonly measured by evaluating malondialdehyde (MDA) levels in the heart tissue. It was documented that catechin, both after in vivo pretreatment and in vitro application to the perfusion medium, reduced myocardial lipid peroxidation in the I/R-exposed isolated rat hearts, and this was associated with improved cardiac function during reperfusion [82]. Further, several studies documented decreased myocardial lipid peroxidation associated with cardioprotection due to in vivo EGCG treatment in both LAD occlusion-induced MI [74] and ISO-induced MI [112,115]. Similarly, reduced lipid peroxidation by EGCG treatment was demonstrated in in vitro model of cardiac I/R in H9c2 cells [74].

As for the indirect antioxidant effects of catechins in myocardial I/R injury, it has been widely documented that catechins enhance activities and levels of endogenous antioxidant enzymes, including SOD, CAT and GPx. It has been reported that pretreatment with EGCG (10, 20, and 30 mg/kg for 21 days) to ISO-induced MI in rats significantly increased the activities of SOD, CAT, GPx, GRx, and glutathione-S-transferase (GST) in the heart tissue, while the activities of these enzymes were declined significantly in the ISO-induced hearts, and these enzymatic changes were associated with reduced lipid peroxidation and cardioprotection manifested by improved heart ultrastructure [113]. Similarly, elevated levels of SOD, CAT, GPx, and GRx were associated with cardioprotective effects of EPI (20 mg/kg for 21 days) in ISO-induced MI in rats [100,114]. In addition, increased levels of MnSOD were associated with cardioprotective effects of EGCG in diabetic rat hearts exposed to LAD occlusion induced in vivo I/R (30 min/2 h), as well as in H9c2 cells exposed to H/R (2 h/4 h) under hyperglycemic conditions [74].

### 5.2. Role of Redox Signaling Pathways in Effects of Catechins in Myocardial I/R Injury

Modulation of intracellular redox signaling during myocardial I/R significantly contributes to adaptive responses of the heart induced by various cardioprotective interventions, including protective effects of antioxidant treatments. Hypoxia-inducing factor (HIF) pathway and the antioxidant transcription factor nuclear factor erythroid 2-related factor 2 (Nrf2) signaling pathway are among the most important pathways included in cardioprotection against I/R injury [111]. It is suggested that HIF may play a dual role in the I/R; on one side, HIF1-α activation is associated with apoptosis and cell death, suggesting that its inhibition might be beneficial for cell survival [116], on the other side, its activation by hypoxia and translocation into the nucleus may activate genes improving cell survival [117]. As for Nrf2, it is considered a key player of redox regulation in cardiovascular diseases and is fundamental in mediating adaptive responses to OxS [111,118].

Regarding the role of HIF signaling in the cardioprotective effects of catechins in I/R injury, it was shown that HIF1-α was significantly elevated in the heart tissue after cardio-pulmonary bypass (CPB = in vivo LAD occlusion) in domestic pigs. Histological analysis detected a massive increase in HIF1-α positive nuclei, documenting that during ischemia, HIF1-α translocates into the nucleus. This elevation was significantly reduced by EGCG application during CPB, associated with reduced OxS and inflammation but not with improved heart function during CBP [105]. A similar reduction of I/R-induced increase of HIF1-α positive nuclei was reported due to EGCG treatment in an I/R injury model in isolated perfused rabbit hearts, and this was also associated with improved post-ischemic recovery of heart function [84]. These data support the hypothesis of protective effects of HIF inhibition during myocardial I/R, likely leading to the decreased level of OxS within cardiomyocytes; however, additional data are needed to fully clarify the role of HIF signaling in cardioprotection by catechins since the only available data came from two experimental studies performed in the same research group.

As for the role of Nrf2 signaling pathway in the cardioprotective effects of catechins, it is yet poorly understood. Although activation of Nrf2/HO-1 (heme oxygenase 1) pathway by EPI was documented to be associated with protection against cerebral I/R injury [119], and its activation was documented in cardioprotective effects of several other polyphenols [120,121], the role of this redox signaling pathway in effects of catechins in myocardial I/R has, in fact, not been evaluated so far. The only study documenting Nrf2/HO-activation in isolated cardiomyocytes due to catechin-including treatment showed that oolong tea rich in catechins prevented hypoxia-induced cardiomyocyte loss by fortifying Nrf2 signaling [122]. Thus, performing pre-clinical animal studies are urgently needed to reveal the role of Nrf2 signaling in cardioprotective effects of catechins in I/R.

Taken together, it was satisfactorily proven that catechins act as efficient ROS-scavengers and strong antioxidant agents in myocardial I/R injury, performing their effects both by their direct antioxidant actions and indirectly via inducing endogenous antioxidant enzymes, including SOD, CAT, and GPx/GRx. However, the role of main intracellular redox signaling pathways, including HIF and Nrf2/HO-1 pathways, in catechin effects in cardiac I/R has not been sufficiently studied so far, thus, making the role in cardioprotection afforded by catechin treatments inconclusive.

## 6. Role of Mitochondria in Action of Catechins in Myocardial I/R Injury

Mitochondria are crucial organelles for most eukaryotic cells and play an irreplaceable role in cell energy metabolism and overall cellular function, affecting a plethora of functions, including reactive oxygen species production (ROS) and cell death regulation. In addition to the canonical production of ATP and regulation of energy metabolism, mitochondria are also involved in different mechanisms of cell death initiation and execution: on one hand, providing components for regulated processes, such as apoptosis or mitophagy, on the other hand, becoming the culprit itself via disruption of mitochondrial membrane potential (ΔΨm) at inner and outer mitochondrial membranes and formation of mitochondrial permeability transition pore (MPTP). Once the MPTP is formed, it leads to an ionic overload and blebbing of mitochondrial membrane and, eventually, to rupture and cell death associated with inflammation and damage of surrounding structures. Such cell loss, especially in cardiac tissue, is a very unpleasant condition due to its limited regenerative capacity. Thus, manipulation of mitochondrial function becomes a target of interest in many cardioprotective strategies, especially in I/R injury, which is a common factor of many cardiac pathologies [123].

Since catechins and epicatechins are involved in protection against I/R injury, as has been discussed in previous chapters, their potential in affecting mitochondrial function should be taken into consideration. Generally, catechins elicit their I/R-injury-ameliorating effects through the modulation of mitochondrial energy metabolism, salvage of ATP production, decrease in ROS production, calcium overload, and ultimately, attenuation of cell death. All these effects of catechins on mitochondrial function are discussed below.

Cardioprotective effects of catechins have been shown on the level of mitochondrial energy metabolism and antioxidant protection. ROS-scavenging properties of catechins have been demonstrated in various studies [74,93,112]. Indeed, pretreatment with EGCG prevented H_2_O_2_-induced reduction of mitochondrial enzyme expression, involved in redox metabolism, such as aldehyde dehydrogenase 2 (Aldh2), ornithine aminotransferase (Oat), or succinate dehydrogenase (Sdha) in H9c2 cells [124]. In line with these findings, the effects of mitochondrial protection by EGCG treatment were demonstrated also in the model of ISO-induced cardiac damage by restoration of activities or levels of mitochondrial glutathione peroxidase, glutathione reductase, reduced glutathione, isocitrate, succinate, malate, α-ketoglutarate and NADH-dehydrogenases, cytochrome-C-oxidase, and adenosine triphosphate in rat hearts [114,125]. Therefore, normalization of the redox system in mitochondria contributes to cardioprotective effects of catechins in settings of I/R injury as well.

It was observed in isolated mitochondria from the rat hearts undergoing the LAD coronary artery ligation leading to I/R injury and MI that EPI treatment in different concentrations (10^−8^–10^−3^ M) was able to attenuate the mitochondrial swelling induced by calcium overload [98]. Previous studies of this workgroup also reported that pre-treatment with 1 mg/kg EPI in rats undergoing the LAD-induced I/R injury yields cardioprotective effects by decreasing the mitochondrial Ca^2+^ overload and preserving the mitochondrial density 1 h after reperfusion [99]. Indeed, the protective effects of 21-days EGCG pretreatment (30 mg/kg) on calcium-induced mitochondrial damage has been demonstrated in the study of Devika et al., 2008, where such intervention led to a normalization of mitochondrial function and activity of Na^+^/K^+^-ATPase in ISO-induced myocardial damage [125]. Additionally, elevations in [Ca^2+^]m in isolated mitochondria from ischemic hearts of guinea pigs were attenuated by treatment with EGCG or its C-2 epimer GCG in a dose-dependent manner. Such effects were accompanied by normalization of ATP production and decreased activity of caspase-3 [83]. The study of Zhang et al., 2019, has shown that EGCG preconditioning is able to ameliorate I/R injury both in vitro and in vivo, improving cardiac functional parameters while reducing ROS production and the extent of apoptosis. Interestingly, the study of mechanisms behind such cardioprotective effects revealed that pretreatment with EGCG in doses of 6.25 or 25 µM, respectively, is capable of attenuation of mitochondria-associated ROS production, MPTP opening, and prevents the collapse of ΔΨm in H9c2 cardiomyocytes after I/R injury. Authors also implied that improvement of mitochondrial function was associated with reduction of mitochondria-associated apoptotic cell death [72]. All above-mentioned studies suggest that catechin treatment in I/R hearts is capable of attenuating calcium overload in mitochondria, preserving mitochondrial ATP production, and thus, contributing to cardiac injury and cell death to a lesser extent.

Another possible mechanism behind the mitochondrial protection provided by catechins in I/R injury is their ability to normalize an activity of mitochondrial potassium ATP-sensitive channels (mKATP). It has been suggested that a decrease in mKATP activity might induce or potentiate MPTP opening, leading to mitochondrial dysfunction and cell death [126]. Catechin or polyphenol treatment led to reduction in IS and improvement of cardiac function in vivo by potential interaction with mKATP, since the inhibitors of KATP channels, selective or non-selective, abolished such cardioprotection [85,127]. Based on these findings we may hypothesize that catechins interact with activity of mKATP in I/R injury. However, whether these effects are only subsequent to the preservation of ATP production by catechins or they are able to directly modulate the activity of these channels needs to be studied more thoroughly.

Deeper understanding of EGCG-mediated effects on mitochondrial function, including the mitochondrial fusion in I/R injury, is provided by the study of Nan et al., 2019. Authors have focused on the mitochondrial dynamics in I/R injury while EGCG was chosen as the in silico candidate inhibitor of OMA1, a metalloendopeptidase, which is responsible for cleavage of OPA1 (Optic atrophy 1, mitochondrial dynamin-like GTPase) protein upon I/R injury, in conditions with increased OxS. EGCG pretreatment in neonatal mouse cardiac myocytes (NMCMs) attenuated H/R-induced injury by inhibiting OMA1-mediated OPA1 cleavage, resulting in maintaining normal mitochondrial function, preserving cristae structure, and alleviating apoptosis [76].

Taken together, catechins and polyphenolic substances, without any doubt, affect the mitochondrial metabolism and their overall function physiologically, but also in cardiac pathologies such as I/R injury. Many of these mechanisms are attributable to their direct antioxidant or ATP production-preserving properties. However, recent studies suggest that they elicit more selective effects on processes such as mitochondrial fusion/fission or via interaction with mKATP and MPTP opening. Therefore, their cardioprotective potential on the mitochondrial level seems to be an important aspect of their mechanism of action; nonetheless, this issue must be addressed in future studies.

## 7. Role of Protein Kinase Pathways in Effects of Catechins in Cardiac I/R Injury

Several protein kinase pathways have been documented to be associated with cardiac I/R injury, as well as with cardioprotection against this cardiac pathology, including cardioprotection provided by catechins and catechin-enriched foods.

### 7.1. RISK Pathway

Reperfusion injury salvage kinase (RISK) pathway is a major cardioprotective pathway associated with cardioprotective effects obtained by various interventions. This pathway is, in fact, a combination of two parallel cascades, PI3K/Akt pathway and MEK1/ERK1/2 pathway, and its pivotal role in cardioprotection against I/R injury is based on the results documenting either upregulation of proteins involved in the RISK pathway by cardioprotective interventions or lack of cardioprotection using inhibitors of PI3K or ERK in combination with several cardioprotective interventions, e.g., ischemic conditioning (for review see: [128]). Activation of this pathway was documented also in cardioprotection against I/R injury by several exogenously administered compounds, e.g., in our studies we documented PI3K/Akt activation by flavonoid quercetin [129] or by antioxidant hormone oxytocin [130]. Regarding the role of RISK pathway in the cardioprotective effects of catechins, several studies documented its activation due to catechins administration in both in vivo and in vitro models of cardiac I/R injury. In in vitro studies in cardiac-derived cells, it was reported that EGCG, alone or in combination with zinc, improved viability of H9c2 cells after H/R-induced injury, and this was associated with activation of Akt kinase in combined EGCG+Zn treatment. On the other hand, Akt was not activated with EGCG alone, thus, making the role of RISK pathway in EGCG action in this I/R model controversial [77]. The cardioprotective role of PTEN/PI3K/Akt pathway activation was clearly documented in neonatal mouse cardiomyocytes exposed to 12 h anoxia treated with EPI for 1 h before the onset of anoxic conditions. In the study, EPI downregulated the expression of PTEN and enhanced the expression of p-Akt, along with improved cell viability; moreover, this cardioprotection was reversed by the PI3K inhibitor LY294002 [69]. Very recently, catechin enhanced cell viability and relieved apoptosis of H9c2 cells through promoting Akt/GSK-3β activation (in particular, it activated Akt kinase by phosphorylation and inhibited GSK3β by phosphorylation at Ser9) under H/R conditions, and this cardioprotection was abolished by PI3K inhibitor LY294002. Interestingly, catechin-induced activation of Akt/Gsk-3β was reversed by lncRNA MIAT, which also decreased cell viability and promoted cell apoptosis under H/R condition. These results suggest that catechin protects cardiac cells against ischemic damage via Akt/Gsk-3β activation through inhibition of lncRNA MIAT [67]. Involvement of RISK pathway in cardioprotective effects of catechins was reported also in in vivo animal studies of cardiac I/R injury. Panneerselvam et al. [96] found epicatechin-induced upregulation of Akt kinase associated with significant reduction of IS in coronary artery occlusion-induced I/R in mice. In addition, EPI was shown to protect the hearts against in vivo myocardial ischemia induced by ligation of the LAD coronary artery for 7 days via activation of the PTEN/PI3K/Akt pathway in mice. In the study, it was documented that EPI treatment for 10 days prior to the myocardial ischemia operation downregulated PTEN, upregulated p-Akt in the heart tissue, and this was associated with improved cardiac function and decreased myocardial apoptosis, cardiac fibrosis, and myocardial hypertrophy. Moreover, the protective effect of EPI was abolished by co-treatment with PI3K inhibitor LY294002, confirming the crucial role of PI3K/Akt pathway in the protective effects of EPI [69]. Furthermore, EGCG was documented to exert protective effects against myocardial I/R injury through the PI3K/Akt pathway-mediated inhibition of apoptosis in LAD occlusion-induced I/R in rats [104].

### 7.2. JNK/p38-MAPK Pathways

The c-Jun N-terminal kinase (JNK) pathway is one of the major signaling cascades that belongs to the family of mitogen-activated protein kinase (MAPK) pathways. It regulates a number of cellular processes, including cell survival, apoptosis, proliferation, and differentiation. The JNK pathway mostly overlaps with the p38-MAPK pathway. In the heart, JNK pathway, like p38-MAPK pathway, is strongly implicated in cardiac response to stress. When the heart is exposed to OxS or hypertrophic stimuli, JNK is activated via MKK4/7 (mitogen-activated protein kinase kinase 4 and 7) and regulates multiple downstream proteins, including Bcl family proteins, thus, enhancing apoptosis and connexin-43, thus, regulating gap junctions and extracellular matrix proteins, thus, regulating cardiac fibrosis and mitochondrial JNK, thus, regulating mitochondrial function, and by all these mechanisms, the JNK pathway is complexly involved in the development of cardiac hypertrophy (for review see: [131,132]).

In myocardial I/R, the JNK pathway is activated mainly during the reperfusion period and was also reported to participate in ischemic preconditioning (for review see: [133,134]). It can be modulated also by exogenously administered cardioprotective compounds, including natural phenolic compounds [135,136]. As for the role of JNK/p38-MAPK in cardioprotective action of catechins, it has been documented that EGCG infused to the perfusion medium before regional I/R (35 min/2 h) in isolated rat hearts reversed I/R-enhanced p38 phosphorylation and reduced IS [89]. In addition, 2-week oral administration of EGCG to rats significantly decreased the phosphorylation of p38 in the heart tissue, decreased OxS and cardiac apoptosis, and improved cardiac function after I/R injury in isolated hearts [93]. Intravenous administration of EGCG significantly reduced phosphorylation of p38 and JNK and reduced IS in rat hearts exposed to in vivo regional I/R (30 min/2 h) [103]. It has also been reported that EGCG may reduce myocardial fibrosis after in vivo AMI via JNK/AP-1 pathway-mediated attenuation of endoglin expression (a membrane glycoprotein, part of the TGFβ receptor complex) [137] 

The role of JNK pathway has been reported also in cardioprotection by EPI. In C57BL/6 mice subjected to myocardial I/R (30 min/2 h), 10-day EPI treatment prior to I/R, downregulated JNK, along with increased phosphorylation of Src, Akt, and IκBα, and these molecular changes were associated with decreased IS [96]. Additionally, suppression of JNK pathway in combination with suppression of miR-92a has been implicated in the in vitro cardioprotective effect of catechin in hypoxia-challenged H9c2 cells [68]. In addition to the pure compounds, catechin-enriched foods were found to confer cardioprotection via JNK-mediated mechanism, e.g., extract from oolong tea containing high levels of catechins significantly decreased hypoxia-enhanced expression of p-JNK and p-p38 and suppressed cell loss and apoptosis in neonatal cardiomyocytes and H9c2 cells exposed to 24-h hypoxia [122].

Taken together, intracellular protein kinase pathways, mainly activation of PI3K/Akt-mediated signaling and suppression of JNK/p38-MAPK pathway, play an important contributory role in the cardioprotection elicited by catechins and catechin-rich foods against myocardial I/R injury in vivo, ex vivo, and in vitro.

## 8. Role of Apoptosis in Effects of Catechins in Myocardial I/R Injury

Apoptosis, a type of programmed cell death, occurs during development to maintain cell populations in tissues and also plays an essential role in eliminating unwanted, partly damaged cells defective in their function. At the molecular level, this form of cell death requires caspase cascade activation, as these enzymes activate the endonucleases responsible for DNA degradation [138]. Cardiac myocytes undergo apoptosis in response to different extracellular and intracellular stimuli, including hypoxia [68], OxS [139], endoplasmic reticulum stress [140], as well as due to anthracycline treatments [141]. Apoptosis contributes to cell death also in pathological conditions such as cardiac I/R injury [93], myocardial infarction [115], diabetic cardiomyopathy [142], atherosclerosis [143], or heart failure [144].

Myocardial I/R injury triggers many different and crossing signaling pathways and, finally, decides whether the cell survives or dies [145]. The cellular mechanisms that lead to apoptosis consist of a numerous steps cascade, including apoptotic trigger, activation of the initiator caspases (e.g., caspase-8, -9, -10) that act as signaling proteases. They specifically cleave and subsequently step by step activate downstream effector caspases (such as caspase-3, -6, -7), which finally cleave various proteins present in both cytosol and nucleus, leading to apoptosis. It is well established that apoptosis is mainly mediated through two signaling pathways: intrinsic (mitochondrial) and extrinsic (death receptor-dependent) [138]. In the intrinsic cell death pathway, mitochondrial outer membrane permeabilization responds to multiple intracellular stress stimuli (oxidative stress, endoplasmic reticulum stress, excess Ca^2+^ levels, DNA damage, etc.), leading to the release of pro-apoptotic proteins, which contributes to the formation of apoptosome, a complex created of released cytochrome c, cytosolic Apaf-1 (apoptotic protease activating factor-1), and zymogen procaspase-9. This, in turn, leads to proteolytic processing of caspase-9 and activation of effector caspase-3, finally, inducing apoptosis [146,147]. It has been shown that in myocardial I/R, cleaved caspase-3 begins to rise during ischemia, but a significant increase occurs during reperfusion [148]. This apoptotic pathway also includes Bcl-2 family proteins. The interactions between pro-apoptotic proteins Bax, Bak, and anti-apoptotic members of the Bcl-2 family, such as Bcl-2, Bcl-xL, and the BH3-only proteins, are necessary for the apoptotic death process. Thus, a change in the Bax/Bcl-2 ratio of proteins may contribute to the modulation of post-ischemic cardiomyocyte apoptosis. [149]. In addition, the tumor suppressor transcription factor p53, which may induce apoptosis by stimulating the expression of BAX-gene and downregulation of BCL-2, thereby, altering the Bax/Bcl-2 ratio, exerts critical regulatory effects during myocardial I/R injury [150]. Accordingly, overexpression of anti-apoptotic Bcl-2 was shown to decrease the rate of apoptosis and, thus, has a convincing effect on cardiac function in transgenic mice after I/R [151]. The extrinsic apoptotic death pathway is triggered by extracellular signals. They induce apoptosis via stimulation of Fas ligand, tumor necrosis factor (TNF), and TNF-related apoptosis-inducing ligand (TRAIL), which interact with the death receptors. The transmitted death receptor signal induces a cascade of steps, ultimately leading to proteolytic activation of caspase-8 and -10, and, finally, activates effector caspase-3 and -7, thus, inducing apoptosis [152]. Activated and fully maturated caspase-8 also cleaves a cytosolic protein Bid to truncated Bid (tBid), which, in turn, destabilizes the membrane of mitochondria either by interaction with cardiolipin or by activation of the pro-apoptotic proteins Bax/Bak [153]. Finally, intrinsic and extrinsic pathways converge to the execution pathway [138].

OxS, one of the main factors drawn into the development of I/R injury, is known to induce apoptosis. Thus, antioxidant therapy by catechins has been shown to be a useful way of reducing cardiomyocyte apoptosis in experimental conditions in various in vivo, ex vivo, and in vitro models of myocardial I/R injury.

Regarding the anti-apoptotic effects of catechins in vitro, it has been reported that pre-treatment of NRCMs with EGCG (12.5–200 mg/L) prevented cardiomyocyte apoptosis and OxS and increased cell viability [139]. Another study showed that protection of NRCMs from I/R-induced apoptosis due to EGCG treatment was associated with reduced expression of STAT-1 pro-apoptotic target gene, Fas receptor, and reduced caspase-3 [89]. In combination with zinc, EGCG was found to provide anti-apoptotic protection against H/R (3/1 h) injury in H9c2 cells via decreased caspase-3 and PI3K/Akt pathway activation, while zinc potentiates the cardioprotective effect of EGCG [77]. EGCG (20 µM) reduced H/R injury also in H9c2 cells exposed to high glucose, which points to reduced OxS and apoptotic death. EGCG pre-treatment considerably upregulated the levels of SIRT1, indicating a crucial role of SIRT1 in EGCG-mediated protection against I/R under hyperglycemia and suggesting that EGCG may be a beneficial dietary supplement for the prevention of diabetic cardiomyopathy [74]. EGCG (10 μM) was also shown to attenuate apoptosis in H9c2 cells after H/R (4/20 h) via protecting the mitochondrial function, evidenced by stabilization of mitochondrial membrane potential and decreased expressions of mitochondrial damage-related proteins [71]. EGCG also reduced hypoxia-induced self-cleavage of OMA1, a metalloendopeptidase involved in the proteolytic process of the fusion-allowing protein OPA1 in mitochondria, and prevented mitochondrial fragmentation, cytochrome c release, and apoptosis, thus, improving mitochondrial function and ATP production and restoring mitochondrial membrane potential in NMCMs exposed to H/R (6/18 h) [76]. EGCG (6.25 or 25 µM) was also found to prevent mitochondrial damage and cell apoptosis by enhancing Bcl-2 and Bcl-xL, reducing cleaved caspase-9 and -3, and by regulating miR-30a/p53 axis in H9c2 cells exposed to H/R for 6/12 h [72].

Another catechin, EPI (5 µM), was shown to protect NMCMs against 12-h anoxia-induced myocardial apoptosis, likely via enhanced Bcl-2/Bax ratio, reduced cleaved caspase-3, and activation of the PTEN/PI3K/Akt pathway [69]. Furthermore, catechin was shown to protect NRCMs from 16-h hypoxia-induced injury and cell apoptosis, as evidenced by reduced Bax and cleaved caspase-9 and -3, likely through regulating microRNA-92a and JNK pathway [68], and to relieve H/R-induced apoptosis in H9c2 cells through regulating CREB/lncRNA MIAT/Akt/Gsk-3β pathway [67]. Finally, catechin (50 µM) protected NRCMs from H_2_O_2_-induced stress by reducing apoptosis by prevention of DNA fragmentation and cleaved caspase-9 accumulation, along with mitigating intracellular ROS levels and maintaining mitochondrial membrane potential [154].

In ex vivo models of myocardial I/R in an isolated heart, it was documented that oral pretreatment of rats with EGCG (0.1, 1, or 10 mmol/L for 2 weeks) showed reduced activation of p38 and expression of caspase-3 after I/R (30/60min), and this was associated with improved post-ischemic function of isolated hearts, suggesting anti-apoptotic effect of this catechin derivative in I/R [93]. Additionally, direct application of EGCG to perfusion solution 10 min before ischemia and during the whole 120 min reperfusion EGCG raised the levels of anti-apoptotic Bcl-2 and decreased pro-apoptotic Bax levels, resulting in increased Bcl-2/Bax ratio and reduced cleaved caspase-3 in isolated rat hearts. This anti-apoptotic effect of studied catechin EGCG was associated with markedly decreased IS, improved heart function post-I/R, and attenuated LDH release to the effluent, thus, confirming the pivotal role of apoptosis inhibition in cardioprotective effects of EGCG [88]. Similarly, EGCG applied to the perfusion solution 30 min before regional I/R (35 min/120 min) protected cardiac myocytes against I/R-induced apoptotic cell death via reduced phosphorylation of STAT-1 and reduced expression of Fas receptor, a known STAT-1 pro-apoptotic target gene [89]. Finally, a catechin-rich extract from green tea mixed with diet in 0.25% proportion applied to rats for 10 days significantly reduced apoptosis markers in the heart tissue after ex vivo global I/R (20 min/2 h), and this was associated with higher total glutathione and higher activities of the phase 2 enzymes glutamate cysteine ligase and quinone reductase, thus, reducing OxS [155].

Regarding in vivo anti-apoptotic effects of catechins in myocardial I/R, it was found that EGCG pre-treatment (15 mg/kg/day for 7 days) of rats with ISO-induced MI resulted in a significant reduction of apoptotic and necrotic cells accompanied by a decrease of pro-apoptotic proteins Bax, p53, and caspase-9 and -3 and increase of anti-apoptotic Bcl-2. The control rats receiving EGCG showed a decrease in the expression of both caspases in the heart tissue with no effect on Bax/Bcl-2 [115]. Another study noticed that EGCG (10 mg/kg) applied to rats via sublingual intravenous injection 10 min prior to the onset of reperfusion significantly decreased myocardial infarct area, reduced the number of TUNEL-positive (apoptotic) cells, reduced the expression of cleaved caspase-3, and improved heart function after LAD occlusion-induced in vivo I/R (30 min/2 h) [104]. Very similarly, a single dose of EGCG (10 or 20 mg/kg) injected into sublingual veins of rats 30 min before LAD occlusion-induced in vivo I/R (30 min/12h) significantly reduced apoptosis, as evidenced by decreased Bax, cleaved caspase-9 and -3, and increased levels of Bcl-2 and Bcl-xL. This was associated with improved cardiac function after I/R, reduced inflammation and necrosis, and improved arrangement of myocardial cells [72]. The anti-apoptotic effect of EGCG was documented also in STZ-induced diabetic rats exposed to in vivo I/R (30 min/2 h by LAD occlusion) [74], as well as in domestic piglets subjected to in vivo I/R for 90/120 min (by LAD occlusion) in a recent standalone study performed in large animals [105]. These promising data suggest that the anti-apoptotic effect of EGCG in myocardial I/R might represent a crucial mechanism of the cardioprotective effect of catechins also in subjects suffering from metabolic comorbidities and might be potentially transferrable from rodents to large animals and eventually to humans.

Another catechin, procyanidin (50 or 100 mg/kg/day, for 2 weeks p.o.), reduced cardiomyocyte apoptosis after I/R (30 min/120 min) in vivo in a dose-dependent manner in rats. Compared with the ischemic group, procyanidin decreased ROS, the expression of p53, Bax, caspases-9 and -3 and increased Bcl-2 expression and Bcl-2/Bax ratio in catechin-treated rats [156]. In addition, EPI was found to protect against myocardial I/R-induced cardiac injury by reducing myocardial apoptosis, evidenced by enhanced Bcl-2, reduced Bax, and cleaved caspase-3, likely via activation of PTEN/PI3K/Akt pathway. This was associated with improved heart function and reduced cardiac fibrosis and hypertrophy [69].

**Figure 3:**
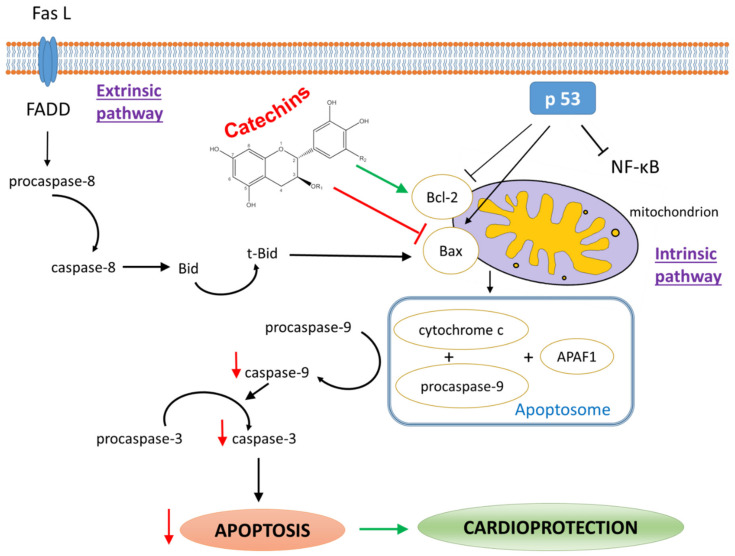
Anti-apoptotic effects of catechins in myocardial I/R injury. Catechins affect several proteins of apoptotic pathways; particularly, they inhibit p53 and enhance Bcl-2/Bax ratio, resulting in reduced caspase-3 activation, thus, reducing apoptosis rate in cardiomyocytes, finally, leading to cardioprotection. Abbreviations: FADD, Fas-associated protein with death domain; NF-kB, nuclear factor kappa-light-chain-enhancer of activated B cells; APAF1, apoptotic protease activating factor 1; Fas L, Fas ligand; tBid, truncated form of Bid; Bid, BH_3_-only protein.

Taken together, reducing apoptosis seems to be a pivotal mechanism of cardioprotection against I/R injury afforded by treatment with catechins (Figure 3). The anti-apoptotic effects of catechins, mainly EGCG, but to a lower extent, also catechin and (−)-epicatechin, were widely documented in various models of myocardial I/R injury in vivo, ex vivo, and in vitro. These anti-apoptotic actions were mediated via modulation of several crossing pathways, such as PTEN/PI3K/Akt, STAT-1, CREB/lncRNA MIAT/Akt/Gsk-3β, JNK, as well as via modulation of mitochondrial function, e.g., by regulation of OMA1/OPA1 proteins. Notably, the anti-apoptotic action of catechins, particularly of EGCG, in cardiac I/R was also proven in large animals and in the presence of metabolic comorbidity, diabetes mellitus. These overall positive and promising results represent a solid pre-clinical base for potential translation of apoptosis-targeting catechin-based cardioprotective approaches into clinical research.

## 9. Role of Non-Coding RNAs in the Cardioprotective Effects of Catechins in I/R Injury

Most of the human genome is intensely transcribed, but less than 2% of these transcripts encode proteins [157]. The rest of the transcripts are non-coding RNAs (ncRNAs), which is a collective term for functional RNA molecules of variable length that do not undergo ribosomal translation into a protein but play important roles in regulating a wide range of cellular processes by repressing or promoting transcription, participating in alternative splicing, or serving as a scaffold for the integration of multiple regulatory proteins [158,159].

MicroRNAs (miRNAs, miRs) are small ncRNAs that are involved in the regulation of gene expression and interfere with virtually all conceivable signaling, metabolic, or regulatory circuits, thereby, contributing to the maintenance of homeostasis. Their levels change due to external stimuli or in the presence of disease, not only in tissues but also in body fluids [160,161,162,163,164]. Numerous miRNAs have been documented to play an important role in myocardial I/R injury and cardioprotection [165]; several of them have also been associated with cardioprotective effects of catechins in I/R.

MiR-30a, a member of the miRNA-30 family, whose members are considered regulators of mitochondrial dynamics and cell death signaling in myocardial I/R injury, as well as in vitro models of OxS, is one of the miRs associated with cardioprotective effects of catechins in I/R. Notably, miR-30a targets p53 protein, which is involved in the process of apoptosis. Involvement of miRNA-30a in cardioprotection provided by catechin derivative EGCG was documented in a combined study comprising rat model of in vivo myocardial I/R injury (30 min/12 h) pretreated with EGCG (20 mg/kg, i.v., 30 min before I/R) and in vitro model of H/R (6/12 h) in H9c2 cells pretreated with EGCG (25 μM). In this study, EGCG mitigated I/R-induced cell apoptosis both in vivo and in vitro, partially preserved heart function and reduced H9c2 cardiomyocyte loss, decreased ROS levels, and improved mitochondrial function. The cardioprotective effect of EGCG was associated with restored miR-30a expression and downregulation of p53. The effect of EGCG was improved by miR-30a mimic and repressed by miR-30a inhibitor. EGCG reversed the expression of mitochondrial apoptosis-related proteins downstream of the miR-30a/p53 pathway, altogether indicating that EGCG may attenuate I/R-induced impairment of mitochondria and apoptosis of myocardial cells through regulation of miR-30a/p53 axis [72]. It has also been documented that exosomes derived from EGCG-treated cardiomyocytes attenuated AMI via modulating miR-30a and decreasing autophagy by transferring this miRNA to cells. Particularly, EGCG (25μM) upregulated miR30a in H9c2 cells subjected to 24-h hypoxic conditions, as well as in the rat I/R model (10 mg/kg EGCG, i.v. for 2 h before 12 h ischemia), and this was associated with reduced pro-apoptotic caspase-3 and Bax/Bcl2 ratio, as well as enhanced p62 and reduced LC3-II and Beclin 1, pointing to reduced autophagy. Thus, cardioprotection elicited by EGCG against myocardial I/R seems to be, at least partially, mediated through anti-apoptotic and anti-autophagic effects via miR-30a induction [75].

MiR-145, which is considered an important modulator of cardiovascular diseases, is another miRNA involved in catechin effects in myocardial I/R. It has been reported that cardioprotective effects of EGCG both in vitro in hypoxia-exposed cultured rat cardiomyocytes (100 μM EGCG, 2 h hypoxia) and in vivo in LAD ligation-induced ischemia in rats (50 mg/kg/day oral EGCG p.o. for 3,5,7,14,28 days after AMI) are, at least in part, mediated via regulation of this miRNA. In hypoxic conditions in vitro, EGCG reversed hypoxia-induced downregulation of miR-145, Wnt3a, and β-catenin expression while it suppressed enhanced expression of disabled-2 (Dab2, a mitogen-responsive adaptor protein, the target gene of miR-145) in cultured rat cardiomyocytes. In line with in vitro data, in vivo EGCG reduced IS of rat hearts after AMI for 28 days similar to in miR-145 dominant or Dab2 siRNA groups, altogether suggesting that EGCG exerts cardioprotection in I/R by targeting miRNA-145 and, consequently, modulating Dab2/Wnt3a/β-catenin pathway [166].

MiR-92a, which influences the JNK pathway, is another miRNA reported to be tightly associated with CVD, and its expression is upregulated in pathophysiological conditions. It has been reported that pretreatment with catechin (100 or 200 μM, 30 min before 16 h hypoxia) protected H9c2 cells against hypoxia-induced miRNA-92a expression increase, and this suppression of miR-92a was associated with improved cell viability and proliferation and reduced apoptosis, as evidenced by reduced Bax, cleaved-caspase-3, and cleaved-caspase-9 expression, and enhanced JNK pathway, as evidenced by an increased ratio of phospho-JNK/total JNK. Moreover, the effect of catechin on JNK stimulation was enhanced by an inhibitor of miR-92a and suppressed by miR-92a mimic. Similar stimulatory/inhibitory effects of miR-92 inhibitor (potentiates beneficial effects of catechin) and miR-92a mimic (suppression of beneficial effects of catechin) were noticed in other parameters, including cell apoptosis, proliferation, and viability. Thus, it is concluded that catechins execute their cardioprotective effect in I/R injury, at least in part, through regulation of miR-92a/JNK signaling [68].

MiR-384 is thought to mediate cardioprotective effects of catechins in I/R via regulation of cardiac autophagy under ischemic conditions. Autophagy is a highly conserved metabolic process that includes changes in the expression of specific proteins, particularly, beclin-1, microtubule-associated protein 1 light chain 3 (LC3), and cathepsin D, necessary for the formation and elongation of the autophagosomal membrane. The next step is maturation into autolysosomes and the final degradation of autolysosomes. It has been documented that I/R upregulates autophagosomes and autolysosomes (in vitro as well as in vivo), while autophagy can be diminished by activating the PI3K/Akt pathway. EGCG pretreatment alleviated I/R-induced autophagy accompanied by increased cell viability and decreased myocardial infarction size in a combined study of in vivo (LAD ligation-induced I/R for 30 min/12 h in rats) and in vitro (H/R for 6 h/12 h in H9c2 cells) models of myocardial I/R injury. While I/R (H/R) was accompanied with a decrease of miRNA-384 expression, pretreatment with EGCG (25 µM) in vitro, as well as in vivo (10 mg/kg), reversed this decrease. I/R-induced autophagy was significantly inhibited by miR-384 overexpression and Beclin-1 knocking down, accompanied by activation of the PI3K/Akt pathway and an enhanced protective effect of EGCG. Moreover, this was abrogated by the PI3K inhibitor. Altogether, cardioprotection against I/R afforded by EGCG might be mediated via regulating microRNA-384-mediated autophagy by downregulating Beclin-1 through activating the PI3K/Akt signaling pathway [73].

Long non-coding RNA—myocardial infarction-associated transcript (LncRNA MIAT), in addition to miRNAs, also plays a regulatory role in the development of many diseases [167,168] and might protect cardiac tissue against apoptosis due to I/R injury [169]. It has been documented that pretreatment of rats with catechin (250 mg/kg/day intragastrically 10 days before I/R (30 min/24 h by LAD occlusion) downregulated lnc MIAT in the myocardial tissue, and this was associated with cardioprotection manifested by improved heart function and reduced infract size post-MI. In addition, pretreatment of H9c2 cells exposed to H/R (6/12 h) with catechin (1–50 μmol/L, 30 min before H/R induction until the end of reoxygenation) attenuated H/R-induced increase of lncRNA MIAT, along with activation of Akt/Gsk-3β pathway, restored mitochondrial membrane potential (MMP), reduced intracellular ROS, and finally, reduced apoptosis, evidenced by reduced cytochrome c release, and improved cell viability. Altogether, the data suggest that catechin protects cardiomyocytes against I/R-induced apoptosis through adjusting CREB/lncRNA MIAT/Akt/Gsk-3β pathway, suggesting an important regulatory role of this ncRNA-related pathway in the cardioprotective effect of catechin in myocardial I/R injury [67].

**Figure 4:**
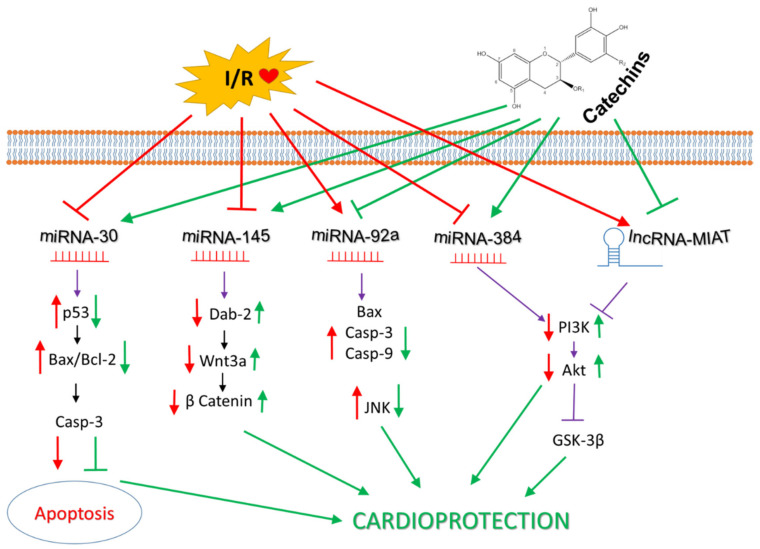
Roles of ncRNAs and associated pathways in cardioprotective effects of catechins in myocardial I/R injury. Abbreviations: Casp-3, caspase 3; Dab-2, disabled homolog-2; Casp-9, caspase-9; PI3K, phosphoinositide 3-kinase; Akt, protein kinase B; GSK-3β, glycogen synthase kinase 3 beta; lncRNA MIAT, long non-coding RNA—myocardial infarction-associated transcript; Labeling: red arrows—changes due to I/R; green arrows—effects of catechins.

Potential regulatory roles of ncRNAs in cardioprotective effects of catechins in myocardial I/R are outlined in Figure 4.

## 10. Dose and Treatment Duration Dependency of Catechins Effects in Myocardial I/R

The important pharmacological aspect of the implication of catechins in myocardial I/R injury is their dosage and treatment duration in relation to their efficiency in preventing myocardial damage in different models of cardiac I/R. Optimization of the dose is a crucial step in research aimed to find the lowest dosage of the compound able to reach maximal cardioprotective effect without detrimental side effects. Optimizing the treatment duration is another important aspect of the efficient implication of catechins into the medical practice; however, it is even more complicated to predict the optimal treatment duration since not only effective dose of catechin derivative should be taken into consideration but also the nature and duration of I/R injury. Thus, the optimal application pattern of the same compound might differ when preventing chronic myocardial injury caused by chronic ischemic heart disease as compared to acute protection, e.g., before planned cardiac surgery. In this chapter, we summarize pre-clinical findings documenting dose/treatment-duration dependency of catechins effects obtained in different in vivo, ex vivo, and in vitro models of myocardial I/R injury.

In pre-clinical in vitro models of cardiac I/R, mostly performed in H9c2 cells or primary cultures of mouse/rat neonatal cardiomyocytes, catechins were administered directly into the culture medium. The most studied catechin derivative in these studies was EGCG, and its effective dose ranged from 6.25 to 100 µM [34,71,74,75,77,89,124,170,171]. The most examined was the acute effect of EGCG (applied 30 min-4 h before I/R) and the most common and effective dose ranged between 10 and 20 µM [71,73,74,75,77,124,171].

The most relevant information about the dose-dependency of effects of any catechin derivative in I/R comes from studies directly comparing different doses of the same compound in the identical experimental settings. Regarding this, only three studies compared different doses of (+)-catechin or EGCG in the same experimental protocol of cardiac I/R in vitro. Akhlagi and Bandy [170] observed that short-term (1 h) pretreatment with 50 μM (+)-catechin leads to 98% improvement in cell viability, while 10 μM concentration of the same compound has non-significant effect in the same experimental settings of H/R. On the other hand, some studies found a cytotoxic effect of EGCG at the 50 µM concentration (the most efficient dose in the above study). In these studies, the most effective concentration of EGCG in preventing I/R injury was 20 µM [72,124]. Only two studies examined the administration of catechins for a longer period (3 days before I/R), using the dose range 5–25 µM [170,172]. However, it is hard to compare the dose efficiency among these studies due to the quite different experimental settings used in these studies, including different timing of H/R protocols, different treatment duration, as well as different types of cell cultures used in the studies.

In studies revealing the effects of catechins in ex vivo I/R injury, catechins have been applied in several different ways: orally mixed with diet or water, by oral gavage, in perfusion buffer, or directly into the heart by infusion pump. The most studied catechins in these studies were EGCG or procyanidins, and less frequently, (+)-catechin and other derivatives. Since procyanidins were applied in the form of various complexes and mixtures, not as pure compounds, they are not included in our further analysis. The most investigated was the acute effect of EGCG applied either before ischemia or at the onset of reperfusion or, eventually, present during the whole I/R protocol. Effective acute dose of EGCG administered by infusion ranged from 1 to 100 µM [85,86,87,89]. Only one study examined EGCG dissolved in a buffer solution during 90 min cardioplegic arrest of rabbit hearts, and its effective dose was 20 µM [84]. The effective doses of another catechin derivative, ECG, dissolved in a buffer solution 4 min before ex vivo I/R or during the initial 4 min of reperfusion in rat hearts were both 100 and 500 ng/mL, while a higher dose (500 ng/mL) provided higher protection [90]. In a long-term study examining the effects of orally given EGCG (in drinking water for 2 weeks) to rats, effective concentrations were both 1 and 10 mM, while paradoxically, the lower dose (1 mM) was more effective than higher one (10 mM) [93]. In another long-term study, the effective dose of EGCG administered by oral gavage for 3 weeks to rats was 200 mg/kg/day [94]. However, it is again hard to compare the dose-dependency of cardioprotective effects among all studies documenting effects of catechins in ex vivo heart I/R due to significantly different experimental protocols used, e.g., acute vs. long-term studies, different mode of compound delivery, and different treatment duration, as well as different animal strains used in the studies. Thus, it is inconclusive to find the optimal dose for ex vivo cardioprotection afforded by catechins.

In the studies of in vivo myocardial I/R, catechins were administered via different ways; the most frequently used were oral gavage and intravenous application. The most studied catechins were EGCG and EPI. The cardioprotective effect of EGCG was demonstrated in both acute and long-term in vivo studies. In acute studies, EGCG was applied either before ischemia or at different times during reperfusion. The effective dose administered intravenously ranged from 10 to 20 mg/kg; the most used dosage was 10 mg/kg [73,75,103,104,105,173]. However, only one study compared two different doses of EGCG (10 vs. 20 mg/kg) in the same experimental protocol and revealed that the higher dose (20 mg/kg) provided a stronger cardioprotective effect than the lower dose (10 mg/kg) [72].

In long-term studies, EGCG was given for 1–3 weeks before MI. The effective dose of EGCG, administered mostly intragastrically, ranged from 10 to 100 mg/kg/day [74,102,112,113,115,125,137,174,175,176]. Again, only one study compared the effectiveness of different doses of EGCG (10, 20, and 30 mg/kg) in the identical experimental protocol. Results showed that all these doses were effective, but the highest dose (30 mg/kg) provided the strongest cardioprotective effect [174].

Dose-dependency of cardioprotective effects of another catechin, EPI, was documented in a study comparing effects of a single (15 min prior to reperfusion) and double dose (15 min prior to reperfusion and 12 h later) of 10 mg/kg EPI (i.v.) on IS in rats exposed to in vivo 45 min LAD occlusion followed by 48-h or 3-week reperfusion. At 48 h of reperfusion, a single dose of EPI reduced IS by 27%, while double dose treatment decreased IS by 80%; and similarly, by 3 weeks reperfusion, a single dose of EPI reduced IS by 28% and double dose treatment further decreased IS by 52%, suggesting an additive cardioprotective effect of double dose of EPI [99]. Similar results were obtained in a study documenting effects of combined treatment with EPI (10 mg/kg, i.v.) and doxocyclin (DOX, 5 mg/kg) in the same experimental settings as in the above-mentioned study (45 min LAD occlusion followed by 48-h or 3-week reperfusion). When applied as a single dose, EPI+DOX reduced IS by 44%, and double dose reduction was 82% at 3 weeks. At 48 h, EPI+DOX treatment reduced MI size by 46%, similarly to 3-week reperfusion; however, the double dose treatment was not studied at 48 h reperfusion [98].

In the long-term studies using daily treatment with EPI (administered repeatedly orally or i.p.), the treatment duration ranged from 2 to 21 days before MI. The effective dose ranged from 1 to 20 mg/kg/day. The cardioprotective effect of EPI was reported already at the dose 1 mg/kg/day when EPI was applied for 10 days prior to MI and continuing until the time of the study termination at 48 h or 3 weeks. Particularly, a significant 50% reduction in IS was found at 48 h of reperfusion but without any significant changes in heart hemodynamics. By 3 weeks of reperfusion, a significant 32% reduction in IS accompanied with sustained hemodynamics and preserved chamber morphometry was observed. Contrary to the 10-day treatment, pretreatment with EPI once per day for only 2 days prior to occlusion and continuing until the time of the study termination (48 h) resulted in no decrease in IS and no changes in hemodynamics [95]. In addition, cardioprotective effects of long-term treatment with EPI in the dose 1 mg/kg/day applied for 10 days before MI were observed in several studies in rats or mice [69,96,97,177]; however, no dose-comparing data were obtained. The only long-term study comparing different doses of EPI (5, 10, and 20 mg/kg/day) was performed in ISO-induced MI rats. The highest dose of EPI (20 mg/kg/day, administered intragastrically for 21 days) provided the strongest cardioprotection, evidenced by significantly lowering the elevated levels of serum creatine kinase-MB compared to the other two doses [100]. Therefore, the highest dose (20 mg/kg body weight) was chosen for further studies in ISO-induced MI [101,114]. In conclusion, the dose of both EGCG and EPI in the in vivo studies ranged usually between 10–30 mg/kg/day, but EPI was found effective already in the dose of 1 mg/kg/day; on the other hand, EGCG has even been used in the dose 100 mg/kg/day. Recently, an even higher dose (250 mg/kg/day) of another compound—catechin—was used for preventing in vivo myocardial I/R in rats [67]. Importantly, a few studies directly pointed to dose-dependency of the cardioprotective effects of catechins in the in vivo myocardial I/R.

**Figure 5:**
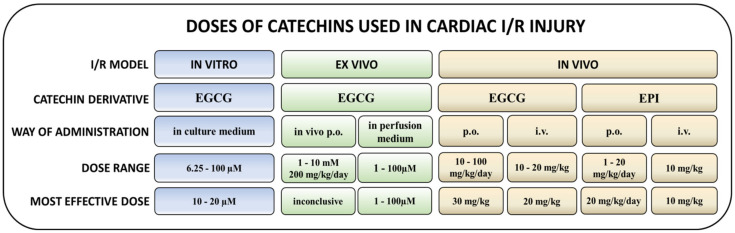
Catechin dosages used in preclinical studies of myocardial I/R injury.

Taken together, although a certain range of efficient dosage of each catechin derivative for all types of myocardial I/R was documented (Figure 5), due to very diverse experimental models and protocols used in studies revealing cardioprotective effects of catechins in myocardial I/R, particularly very variable doses of compounds, different treatment protocols, modes of delivery, and treatment durations, as well as different animal strains used, the exact optimal dosage of a particular catechin derivative cannot be suggested for further pre-clinical investigation, and even less for clinical use of catechins in CVD. Therefore, further investigation focused on the dose-dependency of catechin usage in cardiac I/R injury is necessary; especially, studies using a broad range of different doses of a particular compound in identical experimental protocols documenting effects of catechins in dose-dependent manners are needed to establish optimal dosage of each catechin derivative for the prevention and/or treatment of cardiac I/R injury.

## 11. Translational Gaps in Transferring Cardioprotective Effects of Catechins into Clinics

The crucial aim of biomedical research in the field of cardioprotection is not only bringing new knowledge about potential cardioprotective compounds and interventions but also translation of the pre-clinical knowledge into the clinical practice for the patient’s benefit. However, there are serious translational gaps in the application of cardioprotective strategies into clinics, including those associated with applications of catechins in myocardial I/R injury in human patients. As mentioned earlier in this review, no human studies revealing cardioprotective effects of catechins in myocardial I/R injury in clinical settings, i.e., in ischemic heart disease, myocardial infarction, or cardiac surgery, have been published so far. The absence of clinical studies might be attributed to ethical, technical, and financial difficulties associated with clinical trials but also due to only a limited number of pre-clinical studies documenting cardioprotective effects of catechins in clinically relevant animal models of myocardial I/R injury, i.e., performed in large animals, aged animals, or in the presence of lifestyle-related comorbidities, such as diabetes mellitus, metabolic syndrome, or hypertension. Moreover, in the rare study performed in large animals (domestic pigs), EGCG did not improve cardiac function after in vivo I/R injury induced by LAD coronary artery occlusion even though it reduced markers of myocardial damage [105]. On the other hand, in a rare study performed in STZ-induced diabetic rats, EGCG improved post-ischemic recovery of cardiac function and reduced IS in hearts exposed to in vivo I/R by LAD occlusion, thus, suggesting cardioprotective potential of catechins also in presence of metabolic comorbidities [74]. However, more studies need to be performed in animal models including factors such as ageing, comorbidities, and co-medications to uncover real therapeutic potential of catechins to reduce myocardial I/R injury in human patients. In conclusion, although a large number of pre-clinical studies documented strong cardioprotective effects of various catechins in myocardial I/R in cardiac-derived cell cultures and small animals (exclusively in rodents), there exists still a significant gap in translating this knowledge into clinics due to poorly examined catechin effects in clinically relevant animal models and the lack of any data from human studies.

## 12. Conclusions

As reviewed in this article, numerous pre-clinical studies have revealed a promising therapeutic potential of catechins in myocardial I/R injury. This was widely documented in in vitro, ex vivo, as well as in vivo animal models of cardiac I/R. In vitro models showed strong cardioprotective effects of catechins, mainly EGCG, but also, catechin and EPI manifested in improved cell viability and reduced apoptosis in cardiac-derived cell lines, such as H9c2 cells, as well as in primary cultures of neonatal rodent (rat/mouse) cardiomyocytes exposed to H/R injury for various periods of hypoxia/ischemia. Ex vivo studies performed in Langendorff-perfused isolated rodent hearts, mostly rat hearts, also documented cardioprotective effects of catechins, mainly EGCG, manifested by improved cardiac function and reduced IS post I/R, and these effects were shown after both in vivo and ex vivo application of catechins before maintaining of I/R in isolated hearts. Finally, the vast majority of pre-clinical studies reported cardioprotective effects of catechins, mainly EGCG and EPI, in in vivo models of myocardial ischemia induced by LAD coronary occlusion in rodents. These effects were evidenced by reduced IS and improved cardiac output post-MI. Pre-clinical studies also documented several mechanisms included in cardioprotective effects of catechins, including their antioxidant action directly targeting ROS in the heart tissue under I/R conditions, indirectly via activating endogenous antioxidant enzymes, such as SOD, CAT, and GPx/GRx. Furthermore, catechins were shown to reduce apoptosis, modulate autophagy, and preserve mitochondrial function in I/R via several pathways, including activating PI3K/Akt, inhibition of JNK pathway, as well as via activation of several ncRNA-mediated signaling pathways, such as miR-145, miR-384-5p, miR-30a, miR-92a, and lncRNA MIAT. These results suggest a promising therapeutic potential of catechins in preventing myocardial I/R injury; however, there are also significant translational gaps for implementation of pre-clinical knowledge into clinics since studies performed in clinically relevant animal models reflecting age, sex, comorbidities, or co-medications are rare and human studies are lacking completely. Moreover, the only large animal study documented no improvement of cardiac function after in vivo MI due to EGCG treatment. Thus, more research in clinically relevant animal models of myocardial I/R are needed before research in this area could be translated to human studies and, hopefully, into the clinical practice to improve management of patients suffering from myocardial infarction or chronic ischemic heart disease.

## Figures and Tables

**Table 1:** Effects of catechins on cardiac function, cell viability, and size of infarction in pre-clinical models of I/R.

Improved Parameter	Model of I/R	Mode of Delivery	Compound	Species/Cell Type	References
reduced IS	ex vivo I/R in isolated heart	in perfusion solution	EGCG	rats	[85,86,87,88,89,91]
			ECG	rats	[90]
		orally	EGCG	rats	[94]
	in vivo I/R by LAD occlusion	orally	(−)-epicatechin	rats	[95,97]
				mice	[96]
			Catechin	rats	[67]
			EGCG	rats	[74]
		intravenously	(−)-epicatechin	rats	[98,99]
			EGCG	rats	[73,91,103,104]
	ISO-induced MI	orally	(−)-epicatechin	rats	[101]
improved cardiac function	ex vivo I/R in isolated heart	in perfusion solution	Catechin	rats	[78,79,82]
			Procyanidins	rabbits	[80,81]
			EGCG	guinea pig	[83]
				rabbits	[84]
				rats	[88,89]
			GCG	guinea pig	[83]
		orally	catechin	rats	[82]
			procyanidins	rats	[92]
			EGCG	rats	[93,94]
	in vivo I/R by LAD occlusion	orally	(−)-epicatechin	mice	[69]
			catechin	rats	[67]
			EGCG	rats	[74,102]
		intravenously	EGCG	rats	[72,75,104]
	ISO-induced MI	orally	(−)-epicatechin	rats	[100]
improved cell viability	in vitro H/R	in culture medium	catechin	H9c2	[67,68]
			(−)-epicatechin	NMCMs	[69]
			EGCG	NRCMs	[70]
				H9c2	[72,74,75]

Abbreviations: I/R, ischemia-reperfusion; IS, infarct size; ECG, epicatechin gallate; EGCG, epigallocatechin gallate; LAD, left anterior descending (coronary artery); MI, myocardial infarction; ISO, isoproterenol; NMCMs, neonatal mouse cardiomyocytes; NRCMs, neonatal rat cardiomyocytes.

## References

[1] Tullio F., Angotti C., Perrelli M.-G., Penna C., Pagliaro P. (2013). Redox balance and cardioprotection. Basic Res. Cardiol..

[2] Bartekova M., Barancik M., Dhalla N.S. (2016). Role of Oxidative Stress in Subcellular Defects in Ischemic Heart Disease. Biochemistry of Oxidative Stress.

[3] Ferenczyova K., Kalocayova B., Bartekova M. (2020). Potential Implications of Quercetin and its Derivatives in Cardioprotection. Int. J. Mol. Sci..

[4] Wang W., Kang P.M. (2020). Oxidative Stress and Antioxidant Treatments in Cardiovascular Diseases. Antioxidants.

[5] Cebova M., Pechanova O. (2020). Protective Effects of Polyphenols against Ischemia/Reperfusion Injury. Molecules.

[6] Pandey K.B., Rizvi S.I. (2009). Plant polyphenols as dietary antioxidants in human health and disease. Oxid. Med. Cell. Longev..

[7] Bernatova I. (2018). Biological activities of (-)-epicatechin and (-)-epicatechin-containing foods: Focus on cardiovascular and neuropsychological health. Biotechnol. Adv..

[8] Barteková M., Adameová A., Görbe A., Ferenczyová K., Pecháňová O., Lazou A., Dhalla N.S., Ferdinandy P., Giricz Z. (2021). Natural and synthetic antioxidants targeting cardiac oxidative stress and redox signaling in cardiometabolic diseases. Free Radic. Biol. Med..

[9] Sharifi-Rad M., Pezzani R., Redaelli M., Zorzan M., Imran M., Ahmed Khalil A., Salehi B., Sharopov F., Cho W.C., Sharifi-Rad J. (2020). Preclinical Pharmacological Activities of Epigallocatechin-3-gallate in Signaling Pathways: An Update on Cancer. Molecules.

[10] Manach C., Scalbert A., Morand C., Rémésy C., Jiménez L. (2004). Polyphenols: Food sources and bioavailability. Am. J. Clin. Nutr..

[11] Galleano M., Bernatova I., Puzserova A., Balis P., Sestakova N., Pechanova O., Fraga C.G. (2013). (-)-Epicatechin reduces blood pressure and improves vasorelaxation in spontaneously hypertensive rats by NO-mediated mechanism. IUBMB Life.

[12] Kluknavsky M., Balis P., Puzserova A., Radosinska J., Berenyiova A., Drobna M., Lukac S., Muchova J., Bernatova I. (2016). (−)-Epicatechin Prevents Blood Pressure Increase and Reduces Locomotor Hyperactivity in Young Spontaneously Hypertensive Rats. Oxid. Med. Cell. Longev..

[13] Arazi H., Samami N., Kheirkhah J., Taati B. (2014). The Effect of Three Weeks Green Tea Extract Consumption on Blood Pressure, Heart Rate Responses to a Single Bout Resistance Exercise in Hypertensive Women. High Blood Press. Cardiovasc. Prev..

[14] Rassaf T., Rammos C., Hendgen-Cotta U.B., Heiss C., Kleophas W., Dellanna F., Floege J., Hetzel G.R., Kelm M. (2016). Vasculoprotective Effects of Dietary Cocoa Flavanols in Patients on Hemodialysis: A Double–Blind, Randomized, Placebo–Controlled Trial. Clin. J. Am. Soc. Nephrol..

[15] Pereira T., Bergqvist J., Vieira C., Sveälv B.G., Castanheira J., Conde J. (2019). Randomized study of the effects of cocoa-rich chocolate on the ventricle–arterial coupling and vascular function of young, healthy adults. Nutrition.

[16] Bernatova I., Liskova S. (2021). Mechanisms Modified by (-)-Epicatechin and Taxifolin Relevant for the Treatment of Hypertension and Viral Infection: Knowledge from Preclinical Studies. Antioxidants.

[17] Bae J., Kim N., Shin Y., Kim S.-Y., Kim Y.-J. (2020). Activity of catechins and their applications. Biomed. Dermatol..

[18] Tang G.-Y., Meng X., Gan R.-Y., Zhao C.-N., Liu Q., Feng Y.-B., Li S., Wei X.-L., Atanasov A.G., Corke H. (2019). Health Functions and Related Molecular Mechanisms of Tea Components: An Update Review. Int. J. Mol. Sci..

[19] Ye J.-H., Augustin M.A. (2019). Nano- and micro-particles for delivery of catechins: Physical and biological performance. Crit. Rev. Food Sci. Nutr..

[20] Braicu C., Ladomery M.R., Chedea V.S., Irimie A., Berindan-Neagoe I. (2013). The relationship between the structure and biological actions of green tea catechins. Food Chem..

[21] Botten D., Fugallo G., Fraternali F., Molteni C. (2015). Structural Properties of Green Tea Catechins. J. Phys. Chem. B.

[22] Pastore R.L., Fratellone P. (2006). Potential Health Benefits of Green Tea (*Camellia sinensis*): A Narrative Review. EXPLORE.

[23] Pastoriza S., Mesías M., Cabrera C., Rufián-Henares J.A. (2017). Healthy properties of green and white teas: An update. Food Funct..

[24] Liu Z., Bruins M.E., Ni L., Vincken J.-P. (2018). Green and Black Tea Phenolics: Bioavailability, Transformation by Colonic Microbiota, and Modulation of Colonic Microbiota. J. Agric. Food Chem..

[25] Rashidinejad A., Boostani S., Babazadeh A., Rehman A., Rezaei A., Akbari-Alavijeh S., Shaddel R., Jafari S.M. (2021). Opportunities and challenges for the nanodelivery of green tea catechins in functional foods. Food Res. Int..

[26] Harbowy M.E., Balentine D.A., Davies A.P., Cai Y. (1997). Tea Chemistry. CRC. Crit. Rev. Plant Sci..

[27] Wei K., Wang L., Zhou J., He W., Zeng J., Jiang Y., Cheng H. (2011). Catechin contents in tea (*Camellia sinensis*) as affected by cultivar and environment and their relation to chlorophyll contents. Food Chem..

[28] Hilal Y., Engelhardt U. (2007). Characterisation of white tea–Comparison to green and black tea. J. für Verbraucherschutz und Leb..

[29] Unachukwu U.J., Ahmed S., Kavalier A., Lyles J.T., Kennelly E.J. (2010). White and Green Teas (*Camellia sinensis* var. *sinensis*): Variation in Phenolic, Methylxanthine, and Antioxidant Profiles. J. Food Sci..

[30] Zhao Y., Chen P., Lin L., Harnly J.M., Yu L., Li Z. (2011). Tentative identification, quantitation, and principal component analysis of green pu-erh, green, and white teas using UPLC/DAD/MS. Food Chem..

[31] Dai W., Ruan C., Zhang Y., Wang J., Han J., Shao Z., Sun Y., Liang J. (2020). Bioavailability enhancement of EGCG by structural modification and nano-delivery: A review. J. Funct. Foods.

[32] Gadkari P.V., Balaraman M. (2015). Catechins: Sources, extraction and encapsulation: A review. Food Bioprod. Process..

[33] Chu K.O., Pang C.C.P. (2018). Pharmacokinetics and Disposition of Green Tea Catechins. Pharmacokinetics and Adverse Effects of Drugs-Mechanisms and Risks Factors.

[34] Guo T., Song D., Cheng L., Zhang X. (2019). Interactions of tea catechins with intestinal microbiota and their implication for human health. Food Sci. Biotechnol..

[35] Lipinski C.A., Lombardo F., Dominy B.W., Feeney P.J. (2001). Experimental and computational approaches to estimate solubility and permeability in drug discovery and development settings 1PII of original article: S0169-409X(96)00423-1. The article was originally published in Advanced Drug Delivery Reviews 23 (1997). Adv. Drug Deliv. Rev..

[36] Lambert J.D., Lee M.-J., Lu H., Meng X., Hong J.J.J., Seril D.N., Sturgill M.G., Yang C.S. (2003). Epigallocatechin-3-Gallate Is Absorbed but Extensively Glucuronidated Following Oral Administration to Mice. J. Nutr..

[37] Lu H., Meng X., Yang C.S. (2003). Enzymology of Methylation of Tea Catechins and Inhibition of Catechol-O-methyltransferase by (−)-Epigallocatechin Gallate. Drug Metab. Dispos..

[38] Lu H., Meng X., Li C., Sang S., Patten C., Sheng S., Hong J., Bai N., Winnik B., Ho C.-T. (2003). Glucuronides of Tea Catechins: Enzymology of Biosynthesis and Biological Activities. Drug Metab. Dispos..

[39] Zou L., Peng S., Liu W., Gan L., Liu W., Liang R., Liu C., Niu J., Cao Y., Liu Z. (2014). Improved in vitro digestion stability of (−)-epigallocatechin gallate through nanoliposome encapsulation. Food Res. Int..

[40] Tenore G.C., Campiglia P., Giannetti D., Novellino E. (2015). Simulated gastrointestinal digestion, intestinal permeation and plasma protein interaction of white, green, and black tea polyphenols. Food Chem..

[41] Law F.C.P., Yao M., Bi H.-C., Lam S. (2017). Physiologically based pharmacokinetic modeling of tea catechin mixture in rats and humans. Pharmacol. Res. Perspect..

[42] Nakagawa K., Okuda S., Miyazawa T. (1997). Dose-dependent Incorporation of Tea Catechins, (−)-Epigallocatechin-3-gallate and (−)-Epigallocatechin, into Human Plasma. Biosci. Biotechnol. Biochem..

[43] Lin L.-C., Wang M.-N., Tseng T.-Y., Sung J.-S., Tsai T.-H. (2007). Pharmacokinetics of (−)-Epigallocatechin-3-gallate in Conscious and Freely Moving Rats and Its Brain Regional Distribution. J. Agric. Food Chem..

[44] Catterall F., King L.J., Clifford M.N., Ioannides C. (2003). Bioavailability of dietary doses of 3 H-labelled tea antioxidants (+)-catechin and (-)-epicatechin in rat. Xenobiotica.

[45] Warden B.A., Smith L.S., Beecher G.R., Balentine D.A., Clevidence B.A. (2001). Catechins Are Bioavailable in Men and Women Drinking Black Tea throughout the Day. J. Nutr..

[46] Narumi K., Sonoda J.-I., Shiotani K., Shigeru M., Shibata M., Kawachi A., Tomishige E., Sato K., Motoya T. (2014). Simultaneous detection of green tea catechins and gallic acid in human serum after ingestion of green tea tablets using ion-pair high-performance liquid chromatography with electrochemical detection. J. Chromatogr. B.

[47] Ishii S., Kitazawa H., Mori T., Kirino A., Nakamura S., Osaki N., Shimotoyodome A., Tamai I. (2019). Identification of the Catechin Uptake Transporter Responsible for Intestinal Absorption of Epigallocatechin Gallate in Mice. Sci. Rep..

[48] Hong J., Lambert J.D., Lee S.-H., Sinko P.J., Yang C.S. (2003). Involvement of multidrug resistance-associated proteins in regulating cellular levels of (−)-epigallocatechin-3-gallate and its methyl metabolites. Biochem. Biophys. Res. Commun..

[49] Jodoin J., Demeule M., Béliveau R. (2002). Inhibition of the multidrug resistance P-glycoprotein activity by green tea polyphenols. Biochim. Biophys. Acta-Mol. Cell Res..

[50] Song Q., Li D., Zhou Y., Yang J., Yang W., Zhou G., Wen J. (2014). Enhanced uptake and transport of (+)-catechin and (-)-epigallocatechin gallate in niosomal formulation by human intestinal Caco-2 cells. Int. J. Nanomed..

[51] Vaidyanathan J.B., Walle T. (2003). Cellular Uptake and Efflux of the Tea Flavonoid (-)Epicatechin-3-gallate in the Human Intestinal Cell Line Caco-2. J. Pharmacol. Exp. Ther..

[52] Zhang L., Zheng Y., Chow M.S.S., Zuo Z. (2004). Investigation of intestinal absorption and disposition of green tea catechins by Caco-2 monolayer model. Int. J. Pharm..

[53] Chan K.Y., Zhang L., Zuo Z. (2010). Intestinal efflux transport kinetics of green tea catechins in Caco-2 monolayer model. J. Pharm. Pharmacol..

[54] Dueñas M., Cueva C., Muñoz-González I., Jiménez-Girón A., Sánchez-Patán F., Santos-Buelga C., Moreno-Arribas M., Bartolomé B. (2015). Studies on Modulation of Gut Microbiota by Wine Polyphenols: From Isolated Cultures to Omic Approaches. Antioxidants.

[55] Monagas M., Urpi-Sarda M., Sánchez-Patán F., Llorach R., Garrido I., Gómez-Cordovés C., Andres-Lacueva C., Bartolomé B. (2010). Insights into the metabolism and microbial biotransformation of dietary flavan-3-ols and the bioactivity of their metabolites. Food Funct..

[56] Stalmach A., Mullen W., Steiling H., Williamson G., Lean M.E.J., Crozier A. (2010). Absorption, metabolism, and excretion of green tea flavan-3-ols in humans with an ileostomy. Mol. Nutr. Food Res..

[57] Lambert J.D., Sang S., Yang C.S. (2007). Biotransformation of Green Tea Polyphenols and the Biological Activities of Those Metabolites. Mol. Pharm..

[58] Williamson G., Clifford M.N. (2017). Role of the small intestine, colon and microbiota in determining the metabolic fate of polyphenols. Biochem. Pharmacol..

[59] Fan F.-Y., Shi M., Nie Y., Zhao Y., Ye J.-H., Liang Y.-R. (2016). Differential behaviors of tea catechins under thermal processing: Formation of non-enzymatic oligomers. Food Chem..

[60] Kohri T., Matsumoto N., Yamakawa M., Suzuki M., Nanjo F., Hara Y., Oku N. (2001). Metabolic fate of (-)-[4-(3)H]epigallocatechin gallate in rats after oral administration. J. Agric. Food Chem..

[61] Takagaki A., Nanjo F. (2010). Metabolism of (−)-Epigallocatechin Gallate by Rat Intestinal Flora. J. Agric. Food Chem..

[62] Lambert J.D., Lee M.-J., Diamond L., Ju J., Hong J., Bose M., Newmark H.L., Yang C.S. (2006). Dose-dependent levels of epigallocatechin-3-gallate in human colon cancer cells and mouse plasma and tissues. Drug Metab. Dispos..

[63] Kohri T., Nanjo F., Suzuki M., Seto R., Matsumoto N., Yamakawa M., Hojo H., Hara Y., Desai D., Amin S. (2001). Synthesis of (−)-[4- 3 H ]Epigallocatechin Gallate and Its Metabolic Fate in Rats after Intravenous Administration. J. Agric. Food Chem..

[64] Takagaki A., Nanjo F. (2013). Catabolism of (+)-Catechin and (−)-Epicatechin by Rat Intestinal Microbiota. J. Agric. Food Chem..

[65] Del Rio D., Calani L., Scazzina F., Jechiu L., Cordero C., Brighenti F. (2010). Bioavailability of catechins from ready-to-drink tea. Nutrition.

[66] Zhu M., Chen Y., Li R.C. (2000). Oral Absorption and Bioavailability of Tea Catechins. Planta Med..

[67] Cong L., Su Y., Wei D., Qian L., Xing D., Pan J., Chen Y., Huang M. (2020). Catechin relieves hypoxia/reoxygenation-induced myocardial cell apoptosis via down-regulating lncRNA MIAT. J. Cell. Mol. Med..

[68] Fang J.-F., Dai J.-H., Ni M., Cai Z.-Y., Liao Y.-F. (2018). Catechin protects rat cardiomyocytes from hypoxia-induced injury by regulating microRNA-92a. Int. J. Clin. Exp. Pathol..

[69] Li J.-W., Wang X.-Y., Zhang X., Gao L., Wang L.-F., Yin X.-H. (2018). (‑)‑Epicatechin protects against myocardial ischemia-induced cardiac injury via activation of the PTEN/PI3K/AKT pathway. Mol. Med. Rep..

[70] Ye J.-X., Wang L., Liang R.-X., Yang B. (2008). Protection and its mechanism of catechin morphon on hypoxia-reoxygenation [corrected] induced injury in myocardial cells. Zhongguo Zhong Yao Za Zhi.

[71] Wang W., Huang X., Shen D., Ming Z., Zheng M., Zhang J. (2018). Polyphenol epigallocatechin-3-gallate inhibits hypoxia/reoxygenation-induced H9C2 cell apoptosis. Minerva Med..

[72] Zhang C., Liao P., Liang R., Zheng X., Jian J. (2019). Epigallocatechin gallate prevents mitochondrial impairment and cell apoptosis by regulating miR-30a/p53 axis. Phytomedicine.

[73] Zhang C., Liang R., Gan X., Yang X., Chen L., Jian J. (2019). MicroRNA-384-5p/Beclin-1 As Potential Indicators For Epigallocatechin Gallate Against Cardiomyocytes Ischemia Reperfusion Injury By Inhibiting Autophagy Via PI3K/Akt Pathway. Drug Des. Devel. Ther..

[74] Wu Y., Xia Z.-Y., Zhao B., Leng Y., Dou J., Meng Q.-T., Lei S.-Q., Chen Z.-Z., Zhu J. (2017). (-)-Epigallocatechin-3-gallate attenuates myocardial injury induced by ischemia/reperfusion in diabetic rats and in H9c2 cells under hyperglycemic conditions. Int. J. Mol. Med..

[75] Zhang C., Gan X., Liang R., Jian J. (2020). Exosomes Derived From Epigallocatechin Gallate-Treated Cardiomyocytes Attenuated Acute Myocardial Infarction by Modulating MicroRNA-30a. Front. Pharmacol..

[76] Nan J., Nan C., Ye J., Qian L., Geng Y., Xing D., Rahman M.S.U., Huang M. (2019). EGCG protects cardiomyocytes against hypoxia-reperfusion injury through inhibition of OMA1 activation. J. Cell Sci..

[77] Zeng X., Tan X. (2015). Epigallocatechin-3-gallate and zinc provide anti-apoptotic protection against hypoxia/reoxygenation injury in H9c2 rat cardiac myoblast cells. Mol. Med. Rep..

[78] Van der Kraaij A.M., Mostert L.J., van Eijk H.G., Koster J.F. (1988). Iron-load increases the susceptibility of rat hearts to oxygen reperfusion damage. Protection by the antioxidant (+)-cyanidanol-3 and deferoxamine. Circulation.

[79] Van der Kraaij A.M., van Eijk H.G., Koster J.F. (1989). Prevention of postischemic cardiac injury by the orally active iron chelator 1,2-dimethyl-3-hydroxy-4-pyridone (L1) and the antioxidant (+)-cyanidanol-3. Circulation.

[80] Facinó R.M., Carini M., Aldini G., Berti F., Rossoni G., Bombardelli E., Morazzoni P. (1996). Procyanidines from Vitis vinifera seeds protect rabbit heart from ischemia/reperfusion injury: Antioxidant intervention and/or iron and copper sequestering ability. Planta Med..

[81] Berti F., Manfredi B., Mantegazza P., Rossoni G. (2003). Procyanidins from Vitis vinifera seeds display cardioprotection in an experimental model of ischemia-reperfusion damage. Drugs Exp. Clin. Res..

[82] Modun D., Music I., Katalinic V., Salamunic I., Boban M. (2003). Comparison of protective effects of catechin applied in vitro and in vivo on ischemia-reperfusion injury in the isolated rat hearts. Croat. Med. J..

[83] Hirai M., Hotta Y., Ishikawa N., Wakida Y., Fukuzawa Y., Isobe F., Nakano A., Chiba T., Kawamura N. (2007). Protective effects of EGCg or GCg, a green tea catechin epimer, against postischemic myocardial dysfunction in guinea-pig hearts. Life Sci..

[84] Salameh A., Schuster R., Dähnert I., Seeger J., Dhein S. (2018). Epigallocatechin Gallate Reduces Ischemia/Reperfusion Injury in Isolated Perfused Rabbit Hearts. Int. J. Mol. Sci..

[85] Song D.-K., Jang Y., Kim J.H., Chun K.-J., Lee D., Xu Z. (2010). Polyphenol (-)-epigallocatechin gallate during ischemia limits infarct size via mitochondrial K(ATP) channel activation in isolated rat hearts. J. Korean Med. Sci..

[86] Kim C.J., Kim J.M., Lee S.R., Jang Y.H., Kim J.H., Chun K.J. (2010). Polyphenol (-)-epigallocatechin gallate targeting myocardial reperfusion limits infarct size and improves cardiac function. Korean J. Anesthesiol..

[87] Lee S.K., Kim J.H., Kim J.S., Jang Y., Kim J., Park Y.H., Chun K.J., Lee M.Y. (2012). Polyphenol (-)-epigallocatechin gallate-induced cardioprotection may attenuate ischemia-reperfusion injury through adenosine receptor activation: A preliminary study. Korean J. Anesthesiol..

[88] Piao C.S., Kim D.-S., Ha K.-C., Kim H.-R., Chae H.-J., Chae S.-W. (2011). The Protective Effect of Epigallocatechin-3 Gallate on Ischemia/Reperfusion Injury in Isolated Rat Hearts: An ex vivo Approach. Korean J. Physiol. Pharmacol..

[89] Townsend P.A., Scarabelli T.M., Pasini E., Gitti G., Menegazzi M., Suzuki H., Knight R.A., Latchman D.S., Stephanou A. (2004). Epigallocatechin-3-gallate inhibits STAT-1 activation and protects cardiac myocytes from ischemia/reperfusion-induced apoptosis. FASEB J..

[90] Qi Y., Yang C., Jiang Z., Wang Y., Zhu F., Li T., Wan X., Xu Y., Xie Z., Li D. (2019). Epicatechin-3-Gallate Signaling and Protection against Cardiac Ischemia/Reperfusion Injury. J. Pharmacol. Exp. Ther..

[91] Tu Q., Jiang Q., Xu M., Jiao Y., He H., He S., Zheng W. (2021). EGCG decreases myocardial infarction in both I/R and MIRI rats through reducing intracellular Ca2+ and increasing TnT levels in cardiomyocytes. Adv. Clin. Exp. Med..

[92] Facino R.M., Carini M., Aldini G., Berti F., Rossoni G., Bombardelli E., Morazzoni P. (1999). Diet enriched with procyanidins enhances antioxidant activity and reduces myocardial post-ischaemic damage in rats. Life Sci..

[93] Yanagi S., Matsumura K., Marui A., Morishima M., Hyon S.-H., Ikeda T., Sakata R. (2011). Oral pretreatment with a green tea polyphenol for cardioprotection against ischemia-reperfusion injury in an isolated rat heart model. J. Thorac. Cardiovasc. Surg..

[94] Potenza M.A., Marasciulo F.L., Tarquinio M., Tiravanti E., Colantuono G., Federici A., Kim J.-A., Quon M.J., Montagnani M. (2007). EGCG, a green tea polyphenol, improves endothelial function and insulin sensitivity, reduces blood pressure, and protects against myocardial I/R injury in SHR. Am. J. Physiol. Endocrinol. Metab..

[95] Yamazaki K.G., Romero-Perez D., Barraza-Hidalgo M., Cruz M., Rivas M., Cortez-Gomez B., Ceballos G., Villarreal F. (2008). Short- and long-term effects of (-)-epicatechin on myocardial ischemia-reperfusion injury. Am. J. Physiol. Heart Circ. Physiol..

[96] Panneerselvam M., Tsutsumi Y.M., Bonds J.A., Horikawa Y.T., Saldana M., Dalton N.D., Head B.P., Patel P.M., Roth D.M., Patel H.H. (2010). Dark chocolate receptors: Epicatechin-induced cardiac protection is dependent on delta-opioid receptor stimulation. Am. J. Physiol. Heart Circ. Physiol..

[97] Yamazaki K.G., Taub P.R., Barraza-Hidalgo M., Rivas M.M., Zambon A.C., Ceballos G., Villarreal F.J. (2010). Effects of (-)-epicatechin on myocardial infarct size and left ventricular remodeling after permanent coronary occlusion. J. Am. Coll. Cardiol..

[98] Ortiz-Vilchis P., Yamazaki K.G., Rubio-Gayosso I., Ramirez-Sanchez I., Calzada C., Romero-Perez D., Ortiz A., Meaney E., Taub P., Villarreal F. (2014). Co-administration of the flavanol (-)-epicatechin with doxycycline synergistically reduces infarct size in a model of ischemia reperfusion injury by inhibition of mitochondrial swelling. Eur. J. Pharmacol..

[99] Yamazaki K.G., Andreyev A.Y., Ortiz-Vilchis P., Petrosyan S., Divakaruni A.S., Wiley S.E., De La Fuente C., Perkins G., Ceballos G., Villarreal F. (2014). Intravenous (-)-epicatechin reduces myocardial ischemic injury by protecting mitochondrial function. Int. J. Cardiol..

[100] Stanely Mainzen Prince P. (2011). A biochemical, electrocardiographic, electrophoretic, histopathological and in vitro study on the protective effects of (-)epicatechin in isoproterenol-induced myocardial infarcted rats. Eur. J. Pharmacol..

[101] Stanely Mainzen Prince P. (2013). (-) Epicatechin prevents alterations in lysosomal glycohydrolases, cathepsins and reduces myocardial infarct size in isoproterenol-induced myocardial infarcted rats. Eur. J. Pharmacol..

[102] He J., Yao J., Sheng H., Zhu J. (2015). Involvement of the dual-specificity tyrosine phosphorylation-regulated kinase 1A-alternative splicing factor-calcium/calmodulin-dependent protein kinase IIδ signaling pathway in myocardial infarction-induced heart failure of rats. J. Card. Fail..

[103] Kim S.J., Li M., Jeong C.W., Bae H.B., Kwak S.H., Lee S.H., Lee H.J., Heo B.H., Yook K.B., Yoo K.Y. (2014). Epigallocatechin-3-gallate, a green tea catechin, protects the heart against regional ischemia-reperfusion injuries through activation of RISK survival pathways in rats. Arch. Pharm. Res..

[104] Xuan F., Jian J. (2016). Epigallocatechin gallate exerts protective effects against myocardial ischemia/reperfusion injury through the PI3K/Akt pathway-mediated inhibition of apoptosis and the restoration of the autophagic flux. Int. J. Mol. Med..

[105] Salameh A., Dhein S., Mewes M., Sigusch S., Kiefer P., Vollroth M., Seeger J., Dähnert I. (2020). Anti-oxidative or anti-inflammatory additives reduce ischemia/reperfusions injury in an animal model of cardiopulmonary bypass. Saudi J. Biol. Sci..

[106] Chatree S., Sitticharoon C., Maikaew P., Pongwattanapakin K., Keadkraichaiwat I., Churintaraphan M., Sripong C., Sririwichitchai R., Tapechum S. (2021). Epigallocatechin gallate decreases plasma triglyceride, blood pressure, and serum kisspeptin in obese human subjects. Exp. Biol. Med..

[107] Hollands W.J., Tapp H., Defernez M., Perez Moral N., Winterbone M.S., Philo M., Lucey A.J., Kiely M.E., Kroon P.A. (2018). Lack of acute or chronic effects of epicatechin-rich and procyanidin-rich apple extracts on blood pressure and cardiometabolic biomarkers in adults with moderately elevated blood pressure: A randomized, placebo-controlled crossover trial. Am. J. Clin. Nutr..

[108] Kirch N., Berk L., Liegl Y., Adelsbach M., Zimmermann B.F., Stehle P., Stoffel-Wagner B., Ludwig N., Schieber A., Helfrich H.-P. (2018). A nutritive dose of pure (-)-epicatechin does not beneficially affect increased cardiometabolic risk factors in overweight-to-obese adults-a randomized, placebo-controlled, double-blind crossover study. Am. J. Clin. Nutr..

[109] Dower J.I., Geleijnse J.M., Gijsbers L., Zock P.L., Kromhout D., Hollman P.C.H. (2015). Effects of the pure flavonoids epicatechin and quercetin on vascular function and cardiometabolic health: A randomized, double-blind, placebo-controlled, crossover trial. Am. J. Clin. Nutr..

[110] Gutiérrez-Salmeán G., Meaney E., Lanaspa M.A., Cicerchi C., Johnson R.J., Dugar S., Taub P., Ramírez-Sánchez I., Villarreal F., Schreiner G. (2016). A randomized, placebo-controlled, double-blind study on the effects of (-)-epicatechin on the triglyceride/HDLc ratio and cardiometabolic profile of subjects with hypertriglyceridemia: Unique in vitro effects. Int. J. Cardiol..

[111] Cadenas S. (2018). ROS and redox signaling in myocardial ischemia-reperfusion injury and cardioprotection. Free Radic. Biol. Med..

[112] Devika P.T., Stanely Mainzen Prince P. (2008). (-)Epigallocatechingallate protects the mitochondria against the deleterious effects of lipids, calcium and adenosine triphosphate in isoproterenol induced myocardial infarcted male Wistar rats. J. Appl. Toxicol..

[113] Devika P.T., Stanely Mainzen Prince P. (2008). Protective effect of (-)-epigallocatechin-gallate (EGCG) on lipid peroxide metabolism in isoproterenol induced myocardial infarction in male Wistar rats: A histopathological study. Biomed. Pharmacother..

[114] Stanely Mainzen Prince P. (2013). (-) Epicatechin attenuates mitochondrial damage by enhancing mitochondrial multi-marker enzymes, adenosine triphosphate and lowering calcium in isoproterenol induced myocardial infarcted rats. Food Chem. Toxicol..

[115] Othman A.I., Elkomy M.M., El-Missiry M.A., Dardor M. (2017). Epigallocatechin-3-gallate prevents cardiac apoptosis by modulating the intrinsic apoptotic pathway in isoproterenol-induced myocardial infarction. Eur. J. Pharmacol..

[116] Guo K., Searfoss G., Krolikowski D., Pagnoni M., Franks C., Clark K., Yu K.T., Jaye M., Ivashchenko Y. (2001). Hypoxia induces the expression of the pro-apoptotic gene BNIP3. Cell Death Differ..

[117] Benizri E., Ginouvès A., Berra E. (2008). The magic of the hypoxia-signaling cascade. Cell. Mol. Life Sci..

[118] Barančík M., Grešová L., Barteková M., Dovinová I. (2016). Nrf2 as a key player of redox regulation in cardiovascular diseases. Physiol. Res..

[119] Shah Z.A., Li R., Ahmad A.S., Kensler T.W., Yamamoto M., Biswal S., Doré S. (2010). The flavanol (-)-epicatechin prevents stroke damage through the Nrf2/HO1 pathway. J. Cereb. Blood Flow Metab..

[120] Xu G., Zhao X., Fu J., Wang X. (2019). Resveratrol increase myocardial Nrf2 expression in type 2 diabetic rats and alleviate myocardial ischemia/reperfusion injury (MIRI). Ann. Palliat. Med..

[121] Tsoumani M., Georgoulis A., Nikolaou P.-E., Kostopoulos I.V., Dermintzoglou T., Papatheodorou I., Zoga A., Efentakis P., Konstantinou M., Gikas E. (2021). Acute administration of the olive constituent, oleuropein, combined with ischemic postconditioning increases myocardial protection by modulating oxidative defense. Free Radic. Biol. Med..

[122] Shibu M.A., Kuo C.-H., Chen B.-C., Ju D.-T., Chen R.-J., Lai C.-H., Huang P.-J., Viswanadha V.P., Kuo W.-W., Huang C.-Y. (2018). Oolong tea prevents cardiomyocyte loss against hypoxia by attenuating p-JNK mediated hypertrophy and enhancing P-IGF1R, p-akt, and p-Badser136 activity and by fortifying NRF2 antioxidation system. Environ. Toxicol..

[123] Halestrap A.P., Clarke S.J., Javadov S.A. (2004). Mitochondrial permeability transition pore opening during myocardial reperfusion--a target for cardioprotection. Cardiovasc. Res..

[124] Chen W.-C., Hsieh S.-R., Chiu C.-H., Hsu B.-D., Liou Y.-M. (2014). Molecular identification for epigallocatechin-3-gallate-mediated antioxidant intervention on the H2O2-induced oxidative stress in H9c2 rat cardiomyoblasts. J. Biomed. Sci..

[125] Devika P.T., Stanely Mainzen Prince P. (2008). (-)Epigallocatechin-gallate (EGCG) prevents mitochondrial damage in isoproterenol-induced cardiac toxicity in albino Wistar rats: A transmission electron microscopic and in vitro study. Pharmacol. Res..

[126] Krolikowski J.G., Bienengraeber M., Weihrauch D., Warltier D.C., Kersten J.R., Pagel P.S. (2005). Inhibition of mitochondrial permeability transition enhances isoflurane-induced cardioprotection during early reperfusion: The role of mitochondrial KATP channels. Anesth. Analg..

[127] Ma H., Huang X., Li Q., Guan Y., Yuan F., Zhang Y. (2011). ATP-dependent potassium channels and mitochondrial permeability transition pores play roles in the cardioprotection of theaflavin in young rat. J. Physiol. Sci..

[128] Rossello X., Yellon D.M. (2018). The RISK pathway and beyond. Basic Res. Cardiol..

[129] Barteková M., Šimončíková P., Fogarassyová M., Ivanová M., Okruhlicová Ľ., Tribulová N., Dovinová I., Barančík M. (2015). Quercetin improves postischemic recovery of heart function in doxorubicin-treated rats and prevents doxorubicin-induced matrix metalloproteinase-2 activation and apoptosis induction. Int. J. Mol. Sci..

[130] Ondrejcakova M., Barancik M., Bartekova M., Ravingerova T., Jezova D. (2012). Prolonged oxytocin treatment in rats affects intracellular signaling and induces myocardial protection against infarction. Gen. Physiol. Biophys..

[131] Javadov S., Jang S., Agostini B. (2014). Crosstalk between mitogen-activated protein kinases and mitochondria in cardiac diseases: Therapeutic perspectives. Pharmacol. Ther..

[132] Ravingerová T., Barancík M., Strnisková M. (2003). Mitogen-activated protein kinases: A new therapeutic target in cardiac pathology. Mol. Cell. Biochem..

[133] Armstrong S.C. (2004). Protein kinase activation and myocardial ischemia/reperfusion injury. Cardiovasc. Res..

[134] Hausenloy D.J., Yellon D.M. (2006). Survival kinases in ischemic preconditioning and postconditioning. Cardiovasc. Res..

[135] Xu T., Wu X., Chen Q., Zhu S., Liu Y., Pan D., Chen X., Li D. (2014). The anti-apoptotic and cardioprotective effects of salvianolic acid a on rat cardiomyocytes following ischemia/reperfusion by DUSP-mediated regulation of the ERK1/2/JNK pathway. PLoS ONE.

[136] Syeda M.Z., Fasae M.B., Yue E., Ishimwe A.P., Jiang Y., Du Z., Yang B., Bai Y. (2019). Anthocyanidin attenuates myocardial ischemia induced injury via inhibition of ROS-JNK-Bcl-2 pathway: New mechanism of anthocyanidin action. Phytother. Res..

[137] Lin C.-M., Chang H., Wang B.-W., Shyu K.-G. (2016). Suppressive effect of epigallocatechin-3-O-gallate on endoglin molecular regulation in myocardial fibrosis in vitro and in vivo. J. Cell. Mol. Med..

[138] Elmore S. (2007). Apoptosis: A Review of Programmed Cell Death. Toxicol. Pathol..

[139] Sheng R., Gu Z., Xie M., Zhou W., Guo C. (2007). EGCG inhibits cardiomyocyte apoptosis in pressure overload-induced cardiac hypertrophy and protects cardiomyocytes from oxidative stress in rats. Acta Pharmacol. Sin..

[140] Zhang S., Cao M., Fang F. (2020). The Role of Epigallocatechin-3-Gallate in Autophagy and Endoplasmic Reticulum Stress (ERS)-Induced Apoptosis of Human Diseases. Med. Sci. Monit..

[141] Saeed N.M., El-Naga R.N., El-Bakly W.M., Abdel-Rahman H.M., ElDin R.A.S., El-Demerdash E. (2015). Epigallocatechin-3-gallate pretreatment attenuates doxorubicin-induced cardiotoxicity in rats: A mechanistic study. Biochem. Pharmacol..

[142] Wu W., Liu X., Han L. (2019). Apoptosis of cardiomyocytes in diabetic cardiomyopathy involves overexpression of glycogen synthase kinase-3β. Biosci. Rep..

[143] Kleinbongard P., Heusch G., Schulz R. (2010). TNFα in atherosclerosis, myocardial ischemia/reperfusion and heart failure. Pharmacol. Ther..

[144] Park M., Shen Y.-T., Gaussin V., Heyndrickx G.R., Bartunek J., Resuello R.R.G., Natividad F.F., Kitsis R.N., Vatner D.E., Vatner S.F. (2009). Apoptosis predominates in nonmyocytes in heart failure. Am. J. Physiol. Circ. Physiol..

[145] Vila-Petroff M., Salas M.A., Said M., Valverde C.A., Sapia L., Portiansky E., Hajjar R.J., Kranias E.G., Mundiña-Weilenmann C., Mattiazzi A. (2007). CaMKII inhibition protects against necrosis and apoptosis in irreversible ischemia–reperfusion injury. Cardiovasc. Res..

[146] Tait S.W.G., Green D.R. (2010). Mitochondria and cell death: Outer membrane permeabilization and beyond. Nat. Rev. Mol. Cell Biol..

[147] Kroemer G., Reed J.C. (2000). Mitochondrial control of cell death. Nat. Med..

[148] Lazou A., Iliodromitis E.K., Cieslak D., Voskarides K., Mousikos S., Bofilis E., Kremastinos D.T. (2006). Ischemic but not mechanical preconditioning attenuates ischemia/reperfusion induced myocardial apoptosis in anaesthetized rabbits: The role of Bcl-2 family proteins and ERK1/2. Apoptosis.

[149] Zhao Z.-Q., Nakamura M., Wang N.-P., Velez D.A., Hewan-Lowe K.O., Guyton R.A., Vinten-Johansen J. (2000). Dynamic Progression of Contractile and Endothelial Dysfunction and Infarct Extension in the Late Phase of Reperfusion. J. Surg. Res..

[150] Xu T., Ding W., Ao X., Chu X., Wan Q., Wang Y., Xiao D., Yu W., Li M., Yu F. (2019). ARC regulates programmed necrosis and myocardial ischemia/reperfusion injury through the inhibition of mPTP opening. Redox Biol..

[151] Brocheriou V., Hagège A.A., Oubenaïssa A., Lambert M., Mallet V.O., Duriez M., Wassef M., Kahn A., Menasché P., Gilgenkrantz H. (2000). Cardiac functional improvement by a human Bcl-2 transgene in a mouse model of ischemia/reperfusion injury. J. Gene Med..

[152] Fesik S.W. (2005). Promoting apoptosis as a strategy for cancer drug discovery. Nat. Rev. Cancer.

[153] Gonzalvez F., Pariselli F., Dupaigne P., Budihardjo I., Lutter M., Antonsson B., Diolez P., Manon S., Martinou J.-C., Goubern M. (2005). tBid interaction with cardiolipin primarily orchestrates mitochondrial dysfunctions and subsequently activates Bax and Bak. Cell Death Differ..

[154] Khurana S., Hollingsworth A., Piche M., Venkataraman K., Kumar A., Ross G.M., Tai T.C. (2014). Antiapoptotic Actions of Methyl Gallate on Neonatal Rat Cardiac Myocytes Exposed to H 2 O 2. Oxid. Med. Cell. Longev..

[155] Akhlaghi M., Bandy B. (2010). Dietary green tea extract increases phase 2 enzyme activities in protecting against myocardial ischemia-reperfusion. Nutr. Res..

[156] Liu D. (2018). Effects of procyanidin on cardiomyocyte apoptosis after myocardial ischemia reperfusion in rats. BMC Cardiovasc. Disord..

[157] (2004). Finishing the euchromatic sequence of the human genome. Nature.

[158] Pertea M. (2012). The Human Transcriptome: An Unfinished Story. Genes.

[159] Karakas D., Ozpolat B. (2021). The Role of LncRNAs in Translation. Non-Coding RNA.

[160] Kura B., Kalocayova B., Devaux Y., Bartekova M. (2020). Potential Clinical Implications of miR-1 and miR-21 in Heart Disease and Cardioprotection. Int. J. Mol. Sci..

[161] Kura B., Szeiffova Bacova B., Kalocayova B., Sykora M., Slezak J. (2020). Oxidative Stress-Responsive MicroRNAs in Heart Injury. Int. J. Mol. Sci..

[162] Kura B., Parikh M., Slezak J., Pierce G.N. (2019). The Influence of Diet on MicroRNAs that Impact Cardiovascular Disease. Molecules.

[163] Viczenczova C., Kura B., Egan Benova T., Yin C., Kukreja R., Slezak J., Tribulova N., Szeiffova Bacova B. (2018). Irradiation-Induced Cardiac Connexin-43 and miR-21 Responses Are Hampered by Treatment with Atorvastatin and Aspirin. Int. J. Mol. Sci..

[164] Viczenczova C., Bacova B.S., Benova T.E., Kura B., Yin C., Weismann P., Kukreja R., Slezak J., Tribulova N. (2016). Myocardial connexin-43 and PKC signalling are involved in adaptation of the heart to irradiation-induced injury: Implication of miR-1 and miR-21. Gen. Physiol. Biophys..

[165] Makkos A., Ágg B., Petrovich B., Varga Z.V., Görbe A., Ferdinandy P. (2021). Systematic review and network analysis of microRNAs involved in cardioprotection against myocardial ischemia/reperfusion injury and infarction: Involvement of redox signalling. Free Radic. Biol. Med..

[166] Lin C.-M., Fang W.-J., Wang B.-W., Pan C.-M., Chua S.-K., Hou S.-W., Shyu K.-G. (2020). (-)-Epigallocatechin Gallate Promotes MicroRNA 145 Expression against Myocardial Hypoxic Injury through Dab2/Wnt3a/β-catenin. Am. J. Chin. Med..

[167] Da C., Gong C.-Y., Nan W., Zhou K.-S., Wu Z.-L., Zhang H.-H. (2020). The role of long non-coding RNA MIAT in cancers. Biomed. Pharmacother..

[168] Zhou H., Wang B., Yang Y., Jia Q., Zhang A., Qi Z., Zhang J. (2019). Long Noncoding RNAs in Pathological Cardiac Remodeling: A Review of the Update Literature. Biomed Res. Int..

[169] Chen L., Zhang D., Yu L., Dong H. (2019). Targeting MIAT reduces apoptosis of cardiomyocytes after ischemia/reperfusion injury. Bioengineered.

[170] Akhlaghi M., Bandy B. (2012). Preconditioning and Acute Effects of Flavonoids in Protecting Cardiomyocytes from Oxidative Cell Death. Oxid. Med. Cell. Longev..

[171] Hsieh S.-R., Hsu C.-S., Lu C.-H., Chen W.-C., Chiu C.-H., Liou Y.-M. (2013). Epigallocatechin-3-gallate-mediated cardioprotection by Akt/GSK-3β/caveolin signalling in H9c2 rat cardiomyoblasts. J. Biomed. Sci..

[172] Chang W.-T., Shao Z.-H., Yin J.-J., Mehendale S., Wang C.-Z., Qin Y., Li J., Chen W.-J., Chien C.-T., Becker L.B. (2007). Comparative effects of flavonoids on oxidant scavenging and ischemia-reperfusion injury in cardiomyocytes. Eur. J. Pharmacol..

[173] Aneja R., Hake P.W., Burroughs T.J., Denenberg A.G., Wong H.R., Zingarelli B. (2004). Epigallocatechin, a Green Tea Polyphenol, Attenuates Myocardial Ischemia Reperfusion Injury in Rats. Mol. Med..

[174] Devika P.T., Stanely Mainzen Prince P. (2010). (—)-Epigallocatechin gallate (EGCG) prevents isoprenaline-induced cardiac toxicity by stabilizing cardiac marker enzymes and membrane-bound ATPases. J. Pharm. Pharmacol..

[175] Devika P.T., Stanely Mainzen Prince P. (2008). Preventive effect of (−)epigallocatechin-gallate (EGCG) on lysosomal enzymes in heart and subcellular fractions in isoproterenol-induced myocardial infarcted Wistar rats. Chem. Biol. Interact..

[176] Devika P.T., Stanely Mainzen Prince P. (2009). Preventive effect of (−)epigallocatechin gallate on lipids, lipoproteins, and enzymes of lipid metabolism in isoproterenol-induced myocardial infarction in rats. J. Biochem. Mol. Toxicol..

[177] Ortiz-Vilchis P., Ortiz-Flores M., Pacheco M., Ramirez-Sanchez I., Moreno-Ulloa A., Vega L., Ortiz A., Villarreal F., Rubio-Gayosso I., Najera N. (2018). The cardioprotective effects of (-)-Epicatechin are mediated through arginase activity inhibition in a murine model of ischemia/reperfusion. Eur. J. Pharmacol..

